# Evolution of Social Insect Polyphenism Facilitated by the Sex Differentiation Cascade

**DOI:** 10.1371/journal.pgen.1005952

**Published:** 2016-03-31

**Authors:** Antonia Klein, Eva Schultner, Helena Lowak, Lukas Schrader, Jürgen Heinze, Luke Holman, Jan Oettler

**Affiliations:** 1 Institut für Zoologie, Universität Regensburg, Regensburg, Germany; 2 Centre of Excellence in Biological Interactions, Department of Biosciences, University of Helsinki, Helsinki, Finland; 3 Research School of Biology, Australian National University, Canberra, Australian Capital Territory, Australia; New York University, UNITED STATES

## Abstract

The major transition to eusociality required the evolution of a switch to canalize development into either a reproductive or a helper, the nature of which is currently unknown. Following predictions from the ‘theory of facilitated variation’, we identify sex differentiation pathways as promising candidates because of their pre-adaptation to regulating development of complex phenotypes. We show that conserved core genes, including the juvenile hormone-sensitive master sex differentiation gene *doublesex* (*dsx*) and a *krüppel homolog 2* (*kr-h2*) with putative regulatory function, exhibit both sex and morph-specific expression across life stages in the ant *Cardiocondyla obscurior*. We hypothesize that genes in the sex differentiation cascade evolved perception of alternative input signals for caste differentiation (i.e. environmental or genetic cues), and that their inherent switch-like and epistatic behavior facilitated signal transfer to downstream targets, thus allowing them to control differential development into morphological castes.

## Author Summary

Division of labor into reproductive queens and helper workers in the societies of ants, bees and wasps is achieved by phenotypic plasticity, which allows individuals to embark on discrete developmental trajectories in response to variable signals. These signals can be genetic, epigenetic or environmental, thereby resembling the extreme variation in signals for sex determination across multicellular animals. We show that common developmental pathways downstream of these input signals, including the conserved sex differentiation gene *doublesex*, regulate sex and caste-specific phenotypic differentiation in the ant species *Cardiocondyla obscurior*. Many different mechanisms of gene regulation have been implicated in controlling caste-specific development in social insects but these all depend on a higher-level genetic switch. We propose that highly conserved hub genes such as *dsx*, which can translate variable input signals into large transcription differences using intermediate-level regulators, are tightly linked with the repeated evolutionary transition to eusociality and caste polyphenism.

## Introduction

The mechanisms underlying the evolution of phenotypic novelty are hotly debated [1–4]. A fundamental question is how small genetic or epigenetic changes can produce a set of simultaneous, complementary phenotypic changes required to generate new adaptive trait combinations. A key prediction of the ‘theory of facilitated variation’ [5] is that regulation acts on evolutionarily conserved switch mechanisms, which then modulate expression of target loci controlling development. This process may facilitate large and complex evolutionary steps because it brings together new combinations of inputs (internal or external stimuli) and outputs (phenotypes) but does not rely on evolution of genes involved in the processes *per se*. Importantly, the reliance of this mode of evolution on conserved genetic and developmental processes increases the likelihood that the outputs will be functionally integrated and thus non-lethal, similar to the ‘two-legged goat effect’, a striking example of phenotypic accommodation in which developmental robustness allows the animal to ‘adapt’ to a previously unselected bipedal lifestyle [6].

Evolution by facilitated variation may be especially important to the origin of developmental polyphenisms in which organisms develop into two or more discrete forms, since polyphenisms typically result from plastic activity of regulatory genes. Additionally, it is likely that regulatory mechanisms controlling one set of polyphenism are pre-adapted to evolve control over newly evolving polyphenisms, for two reasons. Firstly, such mechanisms’ pre-existing sensitivities to variable cues make it more likely that they will evolve the ability to perceive alternative gradients of novel cues, relative to constitutively expressed genes. Secondly, their downstream target genes already show inter-individual variability in expression, and the organism will thus already have evolved alternative responses to this variability.

Gerhart and Kirschner [5] made predictions about the properties of the “core components” which they hypothesize to be the principal drivers of evolutionary novelty, namely that these components should display both robustness and adaptability, as well as exploratory behavior, state-dependent expression and regulatory compartmentation. The sex differentiation pathways exhibit all these properties, making them prime candidates for facilitating the evolution of new forms of polyphenism. Some components of the sex differentiation pathway (such as the *doublesex-mab3* (DM) gene family; [7–9]) are evolutionarily ancient and conserved across diverse metazoa, and thus could potentially be involved in generating novel polyphenism in multiple distantly related taxa. In insects, the sequence of sex determination has been called hourglass-shaped [10], with highly variable input signals and downstream targets, but a small set of conserved core regulatory genes including *transformer* (*tra*) and *doublesex* (*dsx*). *doublesex* is alternatively spliced depending on the presence of an active TRA protein, and its sex-specific isoforms act as transcription factors causing sex-specific gene expression and development through their differential effects on multiple downstream targets [11,12].

The two social insect ‘castes’—queens and workers—differ radically from one another in their developmental environment (e.g. nutritional environment) resulting in differences in size, fecundity, behavior and physiology. Ultimately, the evolution of caste polyphenism thus required concerted evolution of environmental input signals and corresponding developmental responses [13]. Eusociality has evolved at least twice within the Hymenoptera [14], but we presently lack a well-evidenced theory of the genetic mechanisms that allowed caste-specific gene expression to originate. There is increasing evidence that the evolution of polyphenism in ants, bees and wasps was achieved primarily through evolution of regulatory genes, rather than gene content or composition [15–17], but the core components involved are largely unknown. Here, we propose that conserved parts of the sex differentiation cascade, including the transcription factor *doublesex*, evolved sensitivity to new environmental input signals (e.g. nutritional signals), thereby triggering caste-specific gene expression that sends larvae on divergent developmental trajectories. To test this hypothesis, we identified *dsx* and its female- and male-specific isoforms, and measured their expression across life stages in the four discrete morphs (queens, workers, winged males and wingless males) of the ant *Cardiocondyla obscurior*. We find that *dsx* sex-specific isoforms are expressed both sex-specifically and morph-specifically in larvae, pupae and adults. Moreover, ninety other conserved genes with sex-biased expression showed morph-specific expression patterns during larval development, suggesting that co-option of the genes regulating sex differentiation via sex-specific alternative splicing was involved in the origin of morphologically distinct castes.

## Results

### Verification of haplodiploidy

Queens and workers produced from inter-population crosses were heterozygous for diagnostic microsatellite markers, whereas emerging winged and wingless males as well as one sex mosaic individual expressing both male and female characters exclusively carried the maternal alleles (S1 Table). Although single locus complementary sex determination is unlikely because the species regularly engages in inbreeding [18], *C*. *obscurior* appears to use standard haplodiploid sexual reproduction.

### Identification of the functional doublesex paralog of *C*. *obscurior*

The *C*. *obscurior* genome [19] has four paralogs containing the DM domain of *doublesex* (*dsx)* (pfam00751; *Cobs_01393*, *Cobs_07724*, *Cobs_09254* and *Cobs_18158*), representing the ancestral state in holometabolous insects [20]. Sex-specific splice forms are only known from one paralog per species (e.g., in *Apis* [21] and *Nasonia* [20]), and the function of the others is unclear. In *C*. *obscurior*, only *Cobs_01393* was differentially expressed in male and female larval RNAseq data (S2 Table). Moreover, *Cobs_01393* had the highest sequence homology to functional *dsx* in other insects (S1 Fig). Finally, we found that *Cobs_01393* was located within ~79 kb of *prospero*; microsynteny of *prospero* and *dsx* is conserved across the Hymenoptera [20]. We thus conclude that *Cobs_01393* is the functional paralog of *dsx*.

### Sex- and morph-specific expression of *dsx* isoforms

**Fig 1:**
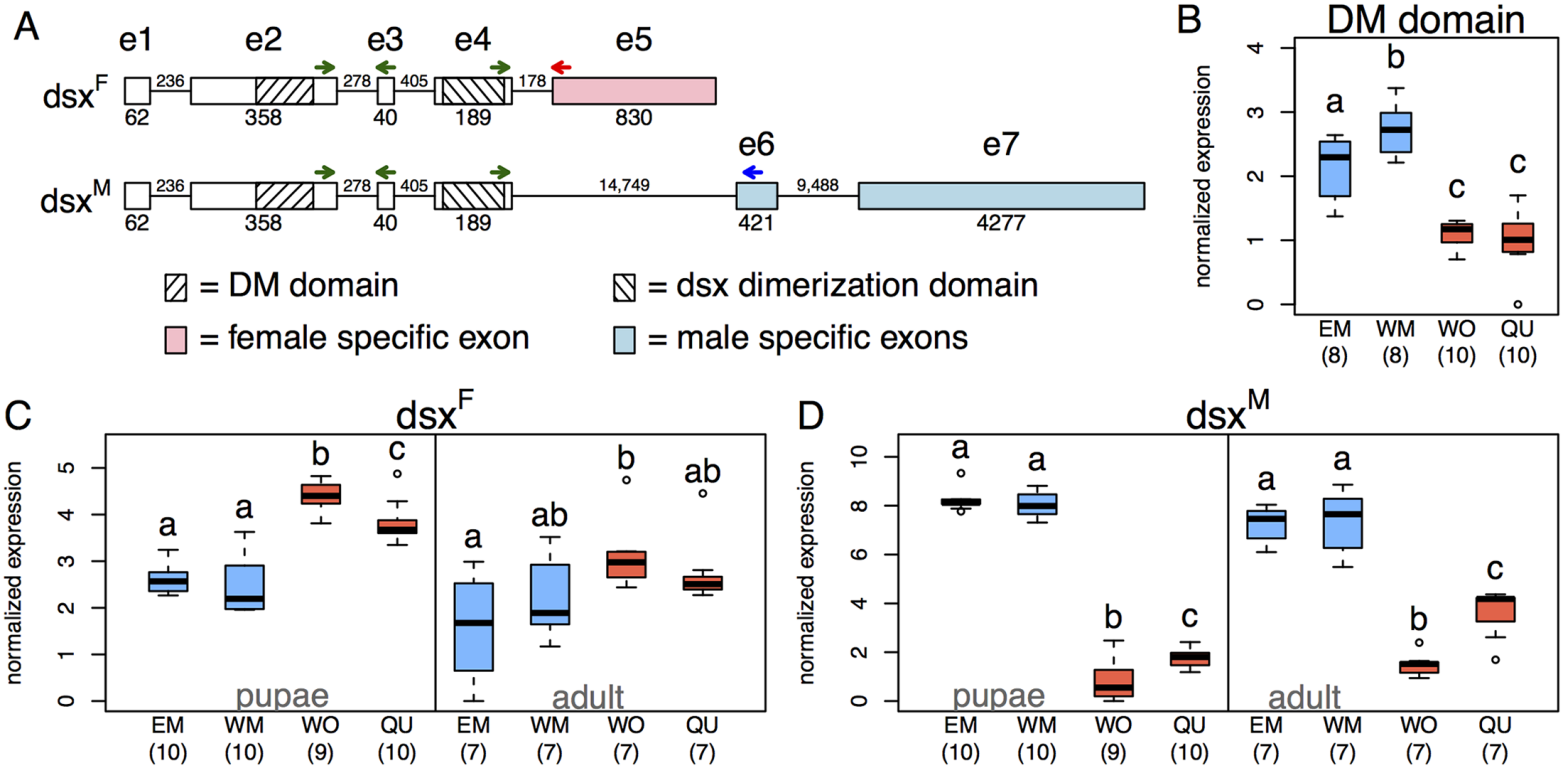
Sex- and morph-specific expression of *dsx* isoforms in the ant *Cardiocondyla obscurior*. (A) Schematic illustration of the sex-specific *doublesex* isoforms *dsx*^F^ and *dsx*^M^. Numbers show sizes of exons and introns in bp. Arrows indicate approximate positions of primers (green: targeting both isoforms, red/blue: specific for *dsx*^F^/*dsx*^M^, respectively; green-exon2: dsx4_for4, green-exon3: dsx4_rev1, green-exon4: 4for, red: F5rev, blue: M5rev). (B-D) Normalized gene expression of *dsx* measured by qPCR in wingless (“ergatoid”) males (EM), winged males (WM), workers (WO) and queens (QU). (B) Expression of the DM domain-encoding exon in adults is higher in males than in females, and higher in winged than in wingless males. (C) *dsx*^F^ is significantly higher expressed in female than in male pupae, whereas *dsx*^M^ is significantly higher expressed in males than in females (D). Worker pupae express significantly more *dsx*^F^ than queen pupae, and adult workers express significantly less *dsx*^M^ than adult queens (C+D). Letters indicate significant differences. Sample sizes are given in parentheses, boxplots show the median, interquartile ranges (IQR) and 1.5 IQR.

We identified the full-length sequence and sex-specific isoforms of the functional paralog of *dsx* using 3’ rapid amplification of cDNA ends (RACE) (S2 Fig). The first four exons are identical in both isoforms. The DM domain (pfam00751) is located in exon 2 and the *dsx* dimerization domain (pfam08828) in exon 4. The female-typical isoform *dsx*^F^ contains one exon specific to *dsx*^F^, whereas the male-typical isoform *dsx*^M^ excludes that exon but includes two others that are absent in *dsx*^F^ (Fig 1A and S3 Table). This splicing pattern, with a shortened female transcript, has been inferred for the fire ant *Solenopsis invicta* [22], and matches *dsx* sex-specific isoforms in *Drosophila melanogaster* and *Apis mellifera*, but not *Nasonia vitripennis* [20]. While the sex-signaling function of *dsx* is conserved across highly divergent lineages, recent evidence shows that *dsx* sequence evolves rapidly [23–25], causing substantial inter-specific variation in *dsx* splicing patterns. A higher level of divergence in *dsx* compared to other DM domain-containing proteins in our phylogenetic analysis confirms this result (S1 Fig).

We designed primers that spanned the exon boundary of the DM domain-containing exon (to measure the overall expression of both isoforms), as well as primers specific to both isoforms for use in RT-qPCR. We found significantly higher expression of the DM domain in adult males (pooling winged males and wingless “ergatoid” males; WM and EM) compared to females (pooling queens and workers; WO and QU) (n_EM_ = 8, n_WM_ = 8, n_WO_ = 10, n_QU_ = 10; Welch two sample t-test: t_25.7_ = -8.7, p<0.001, Fig 1B). Expression of the DM-domain was similar in queens and workers (t-test with Benjamini-Hochberg (BH) correction: p = 0.672), but higher in winged males compared to wingless males (p = 0.009).

We then compared expression of *dsx*^F^ and *dsx*^M^ across all four morphs in pupae (n_EM_ = 10, n_WM_ = 10, n_WO_ = 9, n_QU_ = 10) and adults (n_EM_ = 7, n_WM_ = 7, n_WO_ = 7, n_QU_ = 7; Fig 1C and 1D). We found morph-specific signatures of expression in both life stages for *dsx*^F^ (ANOVA: pupae: F_(3,35)_ = 42.33, p<0.001; adults: F_(3,24)_ = 3.75, p = 0.024) as well as for *dsx*^M^ (Kruskal Wallis rank sum test with df = 3: pupae: X^2^ = 30.2, p<0.001; adults: X^2^ = 22.6, p<0.001). Worker pupae showed significantly higher *dsx*^F^ expression than queen pupae (pairwise t-test with BH correction: p = 0.013) and worker pupae and adults showed significantly lower *dsx*^M^ expression than queen pupae and adults, respectively (Wilcoxon Tests with BH correction: pupae: p = 0.012; adults: p = 0.0014). Neither *dsx*^F^ nor *dsx*^M^ expression differed significantly between the two male morphs (*dsx*^F^: pairwise t-test with BH correction: pupae: p = 0.480, adults: p = 0.277; *dsx*^M^: pairwise Wilcoxon tests with BH correction: pupae: p = 0.481, adults: p = 0.805). However, overall expression of both isoforms was higher in winged compared to wingless males (Fig 1B). Our finding that *dsx* is differentially expressed and alternatively spliced across morphs in pupae and adults suggests that *dsx* might play a role in controlling polyphenic development.

### Tissue specificity of *dsx* splicing in sex mosaics confirms function

To confirm that expression of *dsx* isoforms corresponds with phenotypic tissue differentiation, we used qPCR to analyze *dsx*^M^ and *dsx*^F^ expression in male and female-typical tissues dissected from aberrant “sex mosaic” individuals that express both male and female characters. *C*. *obscurior* sex mosaics are typically laterally separated into female and male halves, indicating that intersexuality is caused by single, early developmental aberrations such as anomalous fertilization events, loss of sex locus expression or inheritance of maternal effects [26–28]. The expression of *dsx*^F^ and *dsx*^M^ was male-typical in male tissue and female-typical in female tissue for all individuals except one, which had similar levels of *dsx*^M^ in both tissue types (S3 Fig). As in previous studies [29,30], we only observed individuals possessing queen and winged male traits, or worker and wingless male traits; other trait combinations were absent (S4 Table), implying that common mechanisms control morph differentiation in males and females.

### Co-option of sex-specific isoforms in larval morph differentiation

We analyzed published RNAseq data [31] from individual early 3^rd^ instar larvae (QU, EM, WM, WO; n = 7 each) on an exon-level with DEXSeq [32]. We found morph-biased expression in each of the seven *dsx* exons, and confirmed sex-specific expression of the DM domain, *dsx*^F^, and *dsx*^M^ in the early 3^rd^ larval stage (S4 Fig and S5 Table). Overall, *dsx* expression was higher in males than in females, and higher in wingless morphs compared to winged morphs (EM > WM, WO > QU).

**Fig 2:**
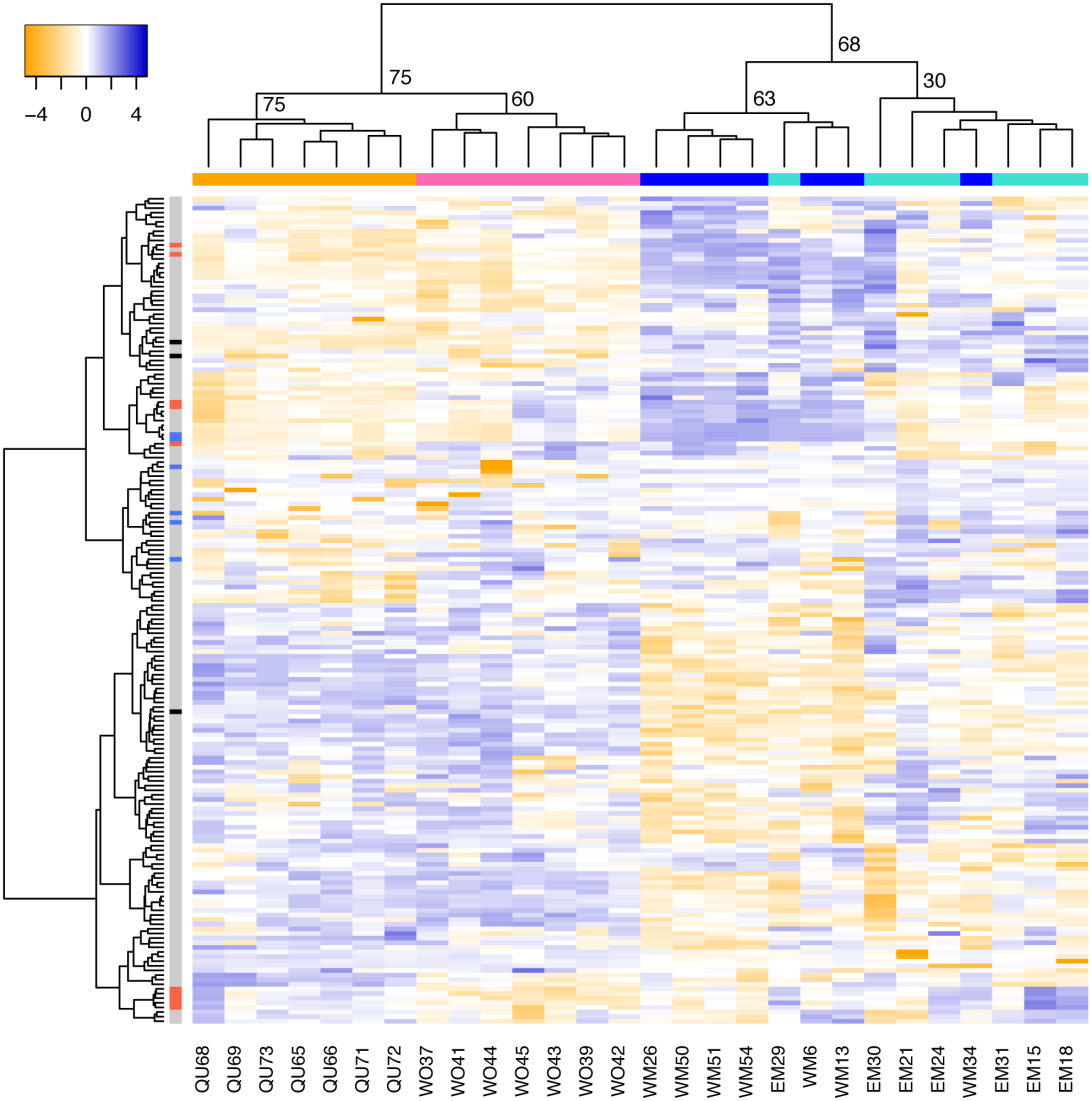
Heat map representing hierarchical clustering of normalized counts for sex-biased exons across the four morphs of the ant *Cardiocondyla obscurior*. Females are clustered into the two castes: workers (WO, pink) and queens (QU, orange). Males also cluster into the two morphs: winged males (WM, dark blue) and wingless males (EM, light blue), with the exception of two samples (WM34, EM29). In the heat map, yellow indicates low expression and blue indicates high expression. Columns represent exons that exhibit sex-biased expression. As revealed by principal component analysis (PCA), exons whose loadings fell into the 10% or 90% quantile on PC 1 and either PC 2 (separating female morphs) or PC 4 (separating male morphs) are indicated in red and blue, respectively. *dsx* exons are highlighted in black (top: exon 7, middle: exon 6, bottom: exon 5). Numbers in the sample-tree show bootstrap values.

We hypothesized that other genes with sex-specific alternative splicing have been similarly co-opted for morph differentiation. Using a conservative false discovery rate of 0.005, DEXSeq analysis identified 179 exons of 91 genes with sex-biased expression (S6 Table). *Dsx* exon 5 (= *dsx*^F^) is ranked 5^th^ among the top 10 differentially expressed exons and exons 6 and 7 (= *dsx*^M^) are the two most significant differentially expressed exons across all samples. To test for co-option of this set of exons into morph differentiation, we performed a hierarchical clustering analysis based on log-transformed exon counts. Queens and workers, as well as winged and wingless males, were clearly separated by the set of sex-biased exons, with the exception of two male samples that clustered with the wrong male morph (bootstrap node support: QU/WO = 75, WM/EM = 68) (Figs 2, S5 and S6 for bootstrap support for all nodes). Because terminal switch points for morph differentiation in male and female larvae may differ [31], misclassification of two male samples (WM34 & EM29) in hierarchical clustering may reflect higher plasticity in males compared to females at this particular developmental stage. Accordingly, in *C*. *obscurior* 3^rd^ instar larvae, more genes are differentially expressed between queens and workers than between winged and wingless males [31].

**Fig 3:**
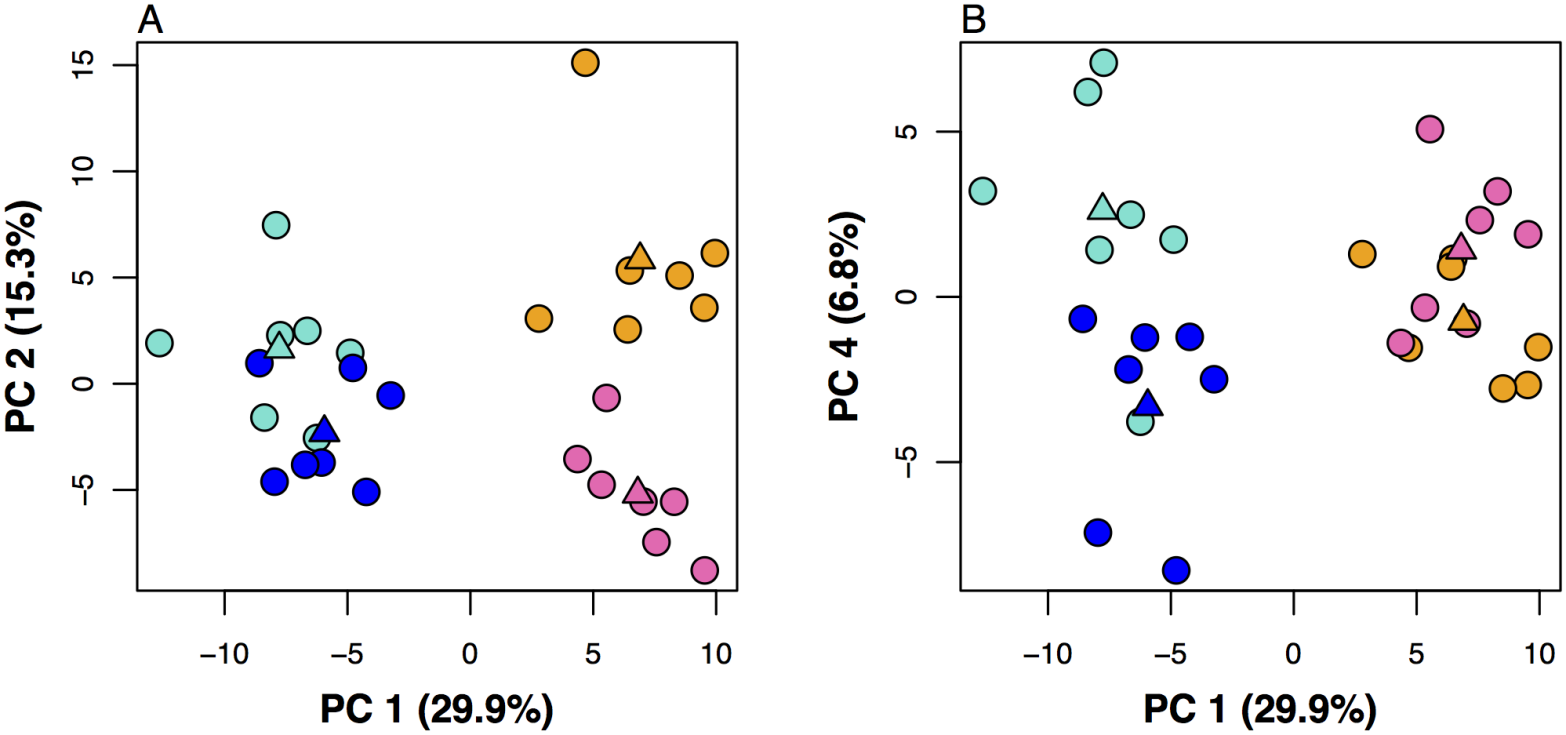
Principal component analysis (PCA) of 179 sex-biased exons in the ant *Cardicondyla obscurior*. PC 1 separates the sexes (A+B), whereas PC 2 separates workers (WO, pink) and queens (QU, orange) (A) and PC 4 separates winged males (WM, dark blue) and wingless males (EM, light blue) (B). Triangles show group centroids.

To identify the sex-biased exons that most strongly affect separation between sexes and morphs, we performed a principal component analysis (PCA) of the 179 normalized exon counts. PC 1 separated sexes (29.9% explained variation), PC 2 (15.3%) and PC 4 (6.8%) separated female and male morphs, respectively (Fig 3; linear discriminant analysis using Wilk’s test on PCs 1, 2 and 4; factor sex: F_(1,28)_ = 95.81, p < 0.001; factor morph: F_(3,28)_ = 27.70, p < 0.001), while PC 3 (7.7%) did not separate between sexes or morphs (linear discriminant analysis using Wilk’s test on PC 3; factor sex: F_(1,28)_ = 0.06, p = 0.80; factor morph: F_(3,28)_ = 1.81, p = 0.17). From the 179 exons, we identified those with the strongest influence on sex (PC 1), female morph (PC 2), and male morph (PC 4) by extracting the exon loadings that fell in either the 10% or 90% quantiles for each PC (S6 Table). Using these lists, we identified *dsx* (replicating the RT-qPCR results) and seven other genes that showed both sex-specific and morph-specific alternative splicing, of which *kr-h2* has a putative transcription factor function (Table 1). All eight genes are conserved across the Insecta, and a Gene Ontology (GO) term enrichment analysis with topGO [33] suggests that they serve basic metabolic and other core functions (S7 Table).

**Table 1:** Eight candidate genes that showed evidence of both sex-specific (sex) and morph-specific (M, F) alternative splicing resulting from a principle component analysis of genes with sex-biased expression. Reciprocal blastp confirms orthology to *D*. *melanogaster* genes.

Gene ID	Alternative splicing	*D*. *melanogaster* gene name	BLASTP evalue/Query/Ident	GO terms (biological process)
*Cobs_01393*	Exon 5,6: M	*doublesex* (*dsx*)	Manually corrected gene model	Regulation of transcription, DNA-templated, sex differentiation
	Exon 7: Sex+M			
*Cobs_03321*	Exon 1,2: F	*mitochondrial trifunctional protein α* (*Mtpalpha*)	0 / 98% / 65%	Limonene catabolic process, beta-alanine metabolic process, benzoate metabolic process, tryptophan metabolic process, isoleucine catabolic process
	Exon 4,5: Sex			
*Cobs_08682*	Exon 1: F	*cyclin-dependent kinase 4* (*Cdk4*)	3e-102 / 55% / 51%	Protein phosphorylation, cell division, serine family amino acid metabolic process
	Exon 3: Sex+M			
*Cobs_04840*	Exon 12,13: Sex	*condensin subunit* (*Cap-D2*)	0 / 72% / 30%	Cell division, mitotic nuclear division, oxidation-reduction process
	Exon 19,20: M			
*Cobs_05728*	Exon 12, 13: M	*ribosomal protein L5* (*RpL5*)	2e-165 / 30% / 76%	Translation, ribosome biogenesis, mismatch repair
	Exon 15,16: Sex			
*Cobs_12024*	Exon 3: Sex	Unnamed (*CG4822*)	2e-152 / 67% / 41%	ATP catabolic process
	Exon 6,9,10:F			
*Cobs_14042*	Exon 1: Sex	*Kruppel homolog 2* (*Kr-h2*)	5e-74 / 90% / 48%	unknown
	Exon 3,4: F			
*Cobs_15702*	Exon 2,6: Sex	*abnormal spindle* (*asp*)	1e-150 / 59% / 36%	Kinetochore organization, centrosome localization, cystoblast division, actin filament reorganization involved in cell cycle

## Discussion

### The role of *dsx* in mediating development

Our study suggests provides evidence that the sex differentiation pathway has been co-opted to control morph-specific development, as we predicted from the theory of facilitated variation. The major candidate gene *dsx* was alternatively spliced in males and females, and differentially expressed between queens and workers and between winged and wingless males. We independently replicated these results using qRT-PCR and RNAseq data from different individuals and life stages. Strikingly, we found that exons showing sex-biased expression were also differentially expressed between morphs, suggesting that *dsx* and other sex-biased genes mediate polyphenism within each of the sexes. The RNAseq analysis conservatively identified eight genes that have sex-specific and morph-specific alternative splicing; all of these genes were evolutionarily conserved and had GO terms associated with basic cellular functions. While *dsx* encodes sex-specific transcription factors and co-ordinates expression of a large number of downstream genes [34], except for a putative role of *kr-h2* (see below) the other genes exhibit no transcription factor function. We confirmed that the sex-specific isoforms of *dsx* correlated with tissue type by analyzing male and female-typical tissue dissected from aberrant sex mosaic individuals. Finally, we reaffirmed that sex mosaics are always either hybrids of a queen and a winged male, or a worker and a wingless male, implying common morph differentiation control mechanisms in both sexes, especially regarding winglessness.

Interestingly, *dsx* has been shown to be a central hub gene involved in generating evolutionary novelty and polyphenism in other taxa. In a butterfly, genetic variation in *dsx* is associated with a heritable female-limited wing color/shape polymorphism, suggesting that *dsx* has been co-opted to control a novel, female-limited trait as well as maintaining its function in sex differentiation [24]. In the genus *Drosophila*, new localizations of *dsx* are thought to have facilitated the evolution of a novel male-limited trait (the sex combs), highlighting how the preexisting sex determination system was co-opted to produce a new polyphenism [35]. In the dung beetle *Onthophagus taurus*, RNAi experiments suggested that variation in *dsx* splicing mediates the difference in the presence of horns between males and females, and also controls a nutritionally dependent, male-limited polyphenism between large-horned and small-horned males [36]. A subsequent study of another horned beetle showed that different *dsx* isoforms control the sensitivity of the mandibles to juvenile hormone (JH), such that male mandibles are stimulated to grow by JH while those of females are not [37]. Thus it appears that *dsx* first evolved to mediate male-limited expression of horns by elevating the sensitivity of male horn tissue to JH [37] and perhaps also the IGF signaling pathway [38], and was then secondarily co-opted to control a nutrition-sensitive, male-limited polyphenism. The beetle *dsx* data are thus highly congruent with the theory of facilitated variation: the male polyphenism evolved using pre-existing genetic switches and developmental mechanisms to link a novel combination of stimuli and outputs (here, larval nutrition and horn phenotype).

Pre- and posttranscriptional genetic tools are not yet well established in ants but there is circumstantial evidence for similar links between *dsx* and JH in *C*. *obscurior*. A previous experiment showed that JH is involved in the development of larvae of both sexes into winged morphs [39], and the present study found differences in *dsx* splicing and expression between winged and wingless morphs. Thus, we speculate that the isoforms of *dsx* may mediate the responsiveness of developing tissues to JH, as hypothesized for beetles [37]. Significant differences in *feminizer* expression between queens and workers in the stingless bee *Melipona* [40], an upstream signal of *dsx* in bees [41], likewise suggests co-option of sex differentiation genes into caste differentiation in bees. There are no homologues of *csd* and *feminizer* in ants, because *csd* evolved in the *Apis* lineage by duplication of *feminizer* [42]. In ants, the closest homologue to *feminizer* is *transformer*. In *C*. *obscurior*, we could not detect morph-specific expression of the two *transformer* paralogues (*tra1*: Cobs_03145 and *tra2*: Cobs_18309), although they were expressed in a sex-specific manner.

In addition to *dsx*, we found a second sex-biased transcript with putative regulatory function. This ortholog to *kr-h2* was alternatively spliced in queen and worker larvae, rendering the *Kruppel homolog* family a promising candidate for modulating plastic responses to the environment. *kr-h2* has structural similarity to the JH-inducible transcription factor *kr-h1* [43], which is involved in the initiation of metamorphosis in other insects [44,45]. *kr-h2*-induced differences in developmental timing may explain why metamorphosis is delayed in queens compared to workers [39], and further points to a link between sex-specific transcription, function in transcriptional regulation, sensitivity to JH, and evolutionary co-option into within-sex polyphenism.

### An extended evo-devo framework for social insect polyphenism

We believe that the hypothesis advanced here, i.e. co-option of sex differentiation pathways into social insect caste polyphenism, is complementary to a previous theory regarding the proximate mechanisms underlying the origin of eusociality, termed the reproductive groundplan hypothesis (RGPH). Based on the ovarian ground plan hypothesis [46], the RGPH posits that eusociality arose via changes in the regulation of pre-existing gene sets relating to reproductive physiology and behavior, for example when genes involved in nest provisioning and brood care began to be expressed in unmated, non-reproductive individuals [47]. Research on the RGPH has stressed the importance of genes with nutrition-sensitive expression in delimiting the queen and worker “genetic toolkits”, in light of evidence that caste fate is nutrition-sensitive [48], that diet preference, reproduction and behavior are pleiotropically linked [49], and that some nutrition-related genes such as *IRS* and *TOR* influence caste fate [50]. Juvenile hormone, which is involved in regulatory feedback loops with some nutrition-related gene networks, has also been linked to caste differences [48,50]—including in our study species *C*. *obscurior* [31,39]—as well as to within-caste polymorphisms (e.g.[51]). Our hypothesis and the RGPH both argue that regulatory evolution caused conserved genes to acquire caste-specific expression. Our hypothesis is distinct in that it explicitly proposes that this regulatory evolution takes place in sex differentiation genes, but leaves the targets of these genes unspecified. By contrast, the RPGH makes predictions about which gene networks produce caste-biased phenotypes (e.g. ovary development, [52]), but makes no prediction regarding the identity of the regulatory sequences controlling these networks. Thus, the hypotheses do not overlap, and both may be correct. Analyses of potential regulatory links between the pathways presented here and those implicated with the RGPH will reveal to what extent they are connected.

### Conclusion

Co-option of conserved genes involved primarily with sex differentiation in novel contexts allows functionally integrated gene networks to produce discrete phenotypes. Together with the horned beetle data reviewed above, our study suggests that core components of the sex differentiation pathway such as *dsx* can produce evolutionary novelty by acting as a switch for nutrition and JH-sensitive growth and development. Although many mechanisms of gene regulation have been implicated in controlling caste-specific development in social insects (e.g. methylation [53], transcription factors [31], small RNAs acting post-transcription [17], RNA editing [54] or structural chromatin modification [55]), all of these depend on some higher-level genetic switch to trigger differential activity. We propose that highly conserved hub genes such as *dsx*, which can translate variable input signals into large transcription differences using intermediate-level regulators, were the most basic mechanism responsible for the repeated evolutionary transition to eusociality and caste polyphenism.

## Material and Methods

### Verification of haplodiploid genetic sex determination

Crosses between five queens of a *C*. *obscurior* population from Japan (JP) and five wingless males of a *C*. *obscurior* population from Brazil (BR) were set up by placing sexual pupae together with some brood and ~20 workers in plaster-filled Petri dishes. Nests were checked twice a week, provided with water, honey and pieces of dead insects and kept at constant conditions (12h 28°C light, 12h 23°C dark). We sampled emerging F1 hybrid QU, WO, EM and WM pupae and extracted DNA from the 10 parental and 71 F1 individuals (23 EM, 3 WM, 22 QU, 22 WO, 1 GY = gynandromorph, for sample sizes per family see S1 Table). Each individual was analyzed at three variable microsatellite loci (Cobs_1.1, Cobs_8.3, Cobs_8.4; for primer sequences see S8 Table). PCRs were performed using the BIO-X-ACT Short Mix (Bioline) and microsatellite analyses were carried out on an ABI PRISM (Applied Biosystems).

### Verification of functional *dsx* and its sex-specific isoforms in *C*. *obscurior*

To find the functional *dsx* ortholog of *C*. *obscurior*, we identified DM domain-containing proteins of *Drosophila melanogaster*, *Nasonia vitripennis*, *Apis mellifera*, *Pogonomyrmex barbatus*, *Acromyrmex echinatior* and *C*. *obscurior* by BLASTp and tBLASTn analyses (S9 Table) and aligned them with MUSCLE [56]. We extracted the DM domain region from the manually corrected alignment (S7 Fig) and built a phylogenetic tree in MEGA [57], applying a WAG+G+I phylogenetic model and bootstrap resampling with 1,000 replicates (S1 Fig).

We reanalyzed previously published RNAseq data of larvae [31]. After removing adapter sequences with cutadapt and performing quality filtration with Trimmomatic, the reads were mapped against the reference genome with tophat2 (v2.0.8) and bowtie2 (v2.1.0) in sensitive mode. We generated count tables with HTseq based on the Cobs1.4 official gene set and used DESeq2 [58] to assess sex-specific expression of the four *dsx* paralogs following size factor normalization.

We applied RACE (Rapid Amplification of cDNA Ends) for identification of *dsx* isoforms. Total RNA was extracted from three females (QU adult, QU pupa, WO pupa) and three wingless males (one pupa, two adults) using the peqGOLD MicroSpin Total RNA Kit (peqlab). Transcription to cDNA was performed with the AffinityScript Multiple Temperature cDNA Synthesis Kit (Agilent Technologies), using the 3’ RACE Adapter GCGAGCACAGAATTAATACGACTCACTATAGGTTTTTTTTTTTTVN. 3’ RACE was performed in a nested PCR using two gene-specific 3’ primers (dsx4_for4, Co_dsx_p3_for, for primer sequences see S8 Table) and the 5’ primer provided in the First Choice RLM-RACE Kit (Ambion). PCRs were performed using the BIO-X-ACT Short Mix (Bioline) with the following protocol: 94°C (3 min), followed by 35 cycles 94°C (30 sec), 60°C (30 sec), 72°C (2 min) and a final elongation of 72°C (7 min). The products were purified with the NucleoSpin Gel and PCR Clean-up (Macherey-Nagel) and Sanger sequenced at LGC Berlin.

### Expression of *dsx* and its isoforms in morphs and sex mosaics with real-time quantitative PCR (qPCR)

Total RNA was extracted from adults (8 EM, 8 WM, 10 QU, 10 WO) using the RNeasy Plus Mini Kit (Qiagen) and transcribed to cDNA using the AffinityScript Multiple Temperature cDNA Synthesis Kit (Agilent Technologies). Expression of the DM domain was quantified by qPCR using the primer pair dsx4_for4/dsx4_rev1 and normalized with two housekeeping genes (RPS2_new, RPL32; see S8 Table for primer sequences).

We further used qPCR to measure isoform-specific *dsx* expression (*dsx*^M^ and *dsx*^F^) in pupae and adults of all four morphs, and in tissue from four sex mosaic pupae. We dissected the head and thoraces of the sex mosaics (for morphological descriptions see S4 Table) laterally into male and female halves and stored male and female tissue parts separately in RNA*later*-ICE (Ambion), resulting in one female and one male sample per individual. We extracted total RNA from 9–10 pupae and seven adults of each of the four morphs, and from the sex mosaic tissue using the peqGOLD MicroSpin Total RNA Kit (peqlab) including a DNA digestion step with the peqGOLD DNase I Digest Kit (peqlab). After cDNA synthesis with iScript cDNA Synthesis Kit (Bio-Rad) we quantified gene expression of *dsx*^F^ and *dsx*^M^ using isoform specific, intron-spanning primers (*dsx*^F^: 4for/F5rev, *dsx*^M^: 4for/M5rev; see Fig 1A for position of primers) and two housekeeping genes (RPS2_new, Y45F10D_JO1). All qPCR reactions were performed in triplicates (repeatability was uniformly high, so we took the mean of the three replicates prior to analysis). Data analysis was carried out according to [59], using the geometric mean of the two housekeeping genes for normalization.

### Testing whether sex-biased exons differ between same-sex morphs

We analyzed published RNAseq data [31] from 3^rd^ star instar larval QU, WO, WM and EM (n = 7 each) and assessed differential exon-specific expression with DEXSeq [32]. Raw reads were trimmed and passed through quality filtration as described in [31] and mapped to the reference genome Cobs1.4 [19] using STAR [60]. We corrected the *dsx* and *tra* gene model using the RACE results for *dsx*, and split the *tra* gene model into two paralogs (*tra1* and *tra2*), as observed in other ants [42,61,62]. For all other genes we used gene models of the Cobs1.4 official gene set. We followed the default workflow of DEXSeq and tested for differential exon usage between males and females based on a false discovery rate of 0.005. In the resulting 179 sex-specific exons, we tested for morph-specific exon profiles using hierarchical clustering (implemented by the R function *hclust* using the ward.D2 method [63]) of pairwise Manhattan distances between log-transformed normalized exon counts. We assessed the support for each node in the cluster analysis using bootstrap resampling with 10,000 replicates using the *pvclust* package in R 3.1.2.

### Identifying genes with sex- and morph-specific exons

We conducted a PCA with normalized exons counts. We visually identified principal components that best separated between sexes (PC 1), female morphs (PC 2) and male morphs (PC 4) and confirmed that these components suffice to separate among sexes and morphs with linear discriminant analysis and subsequent Wilk’s tests in R 3.1.2. Based on loadings of exons on each component, we identified exons that fell in either 10% or 90% quantiles (S6 Table) as those with the strongest influence on PC 1, PC 2 and PC 4. From this list, we extracted only those genes that contained multiple exons with strong influence on both sex (PC 1) and morph (PC 2 and/or PC 4). This yielded a list of eight candidate genes showing alternative splicing between sexes as well as morphs (Table 1).

## Supporting Information

S1 Table Results of microsatellite analyses of F1 individuals from interpopulational crosses. For each family, a Japanese (JP) *Cardiocondyla obscurior* queen was mated with a Brazilian (BR) male. Emerging F1 individuals were genotyped using three population-specific microsatellite markers. This showed that all F1 males (EM = ergatoid, wingless males, WM = winged males) and one gynandromorph (GY) exclusively carried the maternal (JP) allele, whereas emerging females (QU = queens, WO = workers) carried both parental alleles (JP+BR). Sample sizes are given in parenthesis.
(DOCX)

S2 Table Gene expression of the four DM-containing genes of *Cardiocondyla obscurior*. We used previously published RNAseq data and analyzed expression in females vs. males with DESeq2. Only *Cobs_01393* is differentially expressed between the sexes.
(DOCX)

S3 Table Gene structure of *Cardiocondyla obscurior doublesex*. Positions are based on genome version Cobs1.4.
(DOCX)

S4 Table Morphological description of *Cardiocondyla obscurior* sex-mosaics sampled during the course of this study. Sex mosaics are classified as ergatandromorph (E, intersex worker (WO) / ergatoid male (EM)) or gynandromorph (G, intersex queen (QU) / winged male (WM)). Descriptions mainly focus on head morphology, in which differences are most prominent.
(DOCX)

S5 Table Results of statistical tests for normalized count data of expression of *doublesex* exons in RNAseq data. RNAseq data was used to generate per exon count tables for the corrected *dsx* gene model for 3^rd^ instar larvae which were analyzed with Kruskal-Wallis rank sum tests and pairwise Wilcoxon-Test with Benjamini-Hochberg correction.
(DOCX)

S6 Table Loadings of 179 exons on the first four principal components. Bold numbers indicate loadings that fell in the 10% or 90% quantiles for PC1, PC2 & PC4. The 179 sex-biased exons were extracted with DEXseq using a false discovery rate of 0.005.
(DOCX)

S7 Table Gene Ontology (GO) terms for candidate genes associated with female or male morph differentiation. (DOCX)

S8 Table Sequences and targets of primers used in this study, based on genome version Cobs1.4. Primers used for 3’ Rapid amplification of cDNA ends (RACE), in real-time quantitatitive PCR (qPCR) as housekeepers (HK) or targets (T), or for microsatellite analyses (MS).
(DOCX)

S9 Table Overview of DM domain-containing proteins used for phylogenetic tree reconstruction. *Apis mellifera* (Amel), *Nasonia vitripennis* (Nvit), *Cardiocondyla obscurior* (Cobs), *Acromyrmex echinatior* (Aech) and *Pogonomyrmex barbatus* (Pbar) predicted proteins were accessed by BLASTp and tBLASTn analyses on http://hymenopteragenome.org/. Dro*sophila melanogaster* (Dmel) proteins were accessed on http://flybase.org/blast/.
(DOCX)

S1 Fig Phylogenetic tree based on amino acid sequence of the DM domain. Four DM domain-containing paralogous genes each of *Drosophila melanogaster* (Dmel), *Apis mellifera* (Amel), *Nasonia vitripennis* (Nvit), *Acromyrmex echinatior* (Aech), *Pogonomyrmex barbatus* (Pbar) and *Cardiocondyla obscurior* (Cobs) were used. S9 Table contains the complementary sequences. For phylogenetic tree reconstruction we used MEGA, and applied a WAG+G+I phylogenetic model and bootstrap resampling with 1,000 replicates. Numbers show bootstrap support values.
(DOCX)

S2 Fig Gel image of sex-specific *doublesex* splicing variants in *Cardiocondyla obscurior*. Variants were amplified using 3’ RACE (2–4: *dsx*^F^ in females, 6–8: *dsx*^M^ in males).
(DOCX)

S3 Fig Tissue-specific expression of *doublesex* isoforms in sex mosaics of the ant *Cardiocondyla obscurior*. (A) *dsx*^F^ expression is higher in female (red triangles) than male halves (blue squares) of each individual, whereas *dsx*^M^ expression is higher in male than female halves, with one exception (#10) (B).
(DOCX)

S4 Fig Normalized read counts of *doublesex* exons. RNAseq data of larvae of all four morphs (EM = wingless, ergatoid males, WM = winged males, WO = workers, QU = queens, N = 7 each) were analyzed using DEXseq. Female castes show significant differences in expression of exons 3 and 7, male morphs show significant differences in all exons except exon 5. Letters indicate test statistics of pairwise Wilcoxon Tests with Benjamini-Hochberg correction for multiple testing (see S5 Table).
(DOCX)

S5 Fig Cluster dendrogram of larvae based on expression of 179 sex-specific exons. Red values show bootstrap probabilities (bp); blue values show approximately unbiased p-values (au) from pvclust. Workers (WO) and queens (QU) are well separated, whereas one winged male (WM) clusters within the wingless, ergatoid males (EM) and one wingless male clusters within the winged males.
(DOCX)

S6 Fig Cluster dendrogram of 179 sex-specifically expressed exons. Red values show bootstrap probabilities (bp); blue values show approximately unbiased p-values (au) from pvclust.
(DOCX)

S7 Fig Alignment of the amino acid sequences of the DM domain (pfam00751). DM domain-containing proteins of *Drosophila melanogaster* (Dmel), *Apis mellifera* (Amel), *Nasonia vitripennis* (Nvit), *Acromyrmex echinatior* (Aech), *Pogonomyrmex barbatus* (Pbar) and *Cardiocondyla obscurior* (Cobs) were aligned with MUSCLE.
(DOCX)

S1 Data Contains excel files with raw Cq values, 2−ΔΔCq calculation and final R input tabs for dsx DM domain and isoform specific qPCR and for analysis of gynandromorphs. (ZIP)

## References

[1] Conrad B, Antonarakis SE. Gene duplication: a drive for phenotypic diversity and cause of human disease. Annu Rev Genomics Hum Genet. 2007;8: 17–35. 17386002 10.1146/annurev.genom.8.021307.110233

[2] Feschotte C. Transposable elements and the evolution of regulatory networks. Nat Rev Genet. 2008;9: 397–405. 10.1038/nrg2337 18368054 PMC2596197

[3] Tautz D, Domazet-Lošo T. The evolutionary origin of orphan genes. Nat Rev Genet. 2011;12: 692–702. 10.1038/nrg3053 21878963

[4] Blount ZD, Barrick JE, Davidson CJ, Lenski RE. Genomic analysis of a key innovation in an experimental *Escherichia coli* population. Nature. 2012;489: 513–518. 10.1038/nature11514 22992527 PMC3461117

[5] Gerhart J, Kirschner M. The theory of facilitated variation. Proc Natl Acad Sci U S A. 2007;104: 8582–8589. 17494755 10.1073/pnas.0701035104 PMC1876433

[6] West-Eberhard MJ. Developmental Plasticity and Evolution. Oxford University Press; 2003.

[7] Yi W, Zarkower D. Similarity of DNA binding and transcriptional regulation by *Caenorhabditis elegans* MAB-3 and *Drosophila melanogaster* DSX suggests conservation of sex determining mechanisms. Development. 1999;126: 873–881. 9927589 10.1242/dev.126.5.873

[8] Kopp A. Dmrt genes in the development and evolution of sexual dimorphism. Trends Genet. 2012;28: 175–184. 10.1016/j.tig.2012.02.002 22425532 PMC3350790

[9] Matson CK, Zarkower D. Sex and the singular DM domain: insights into sexual regulation, evolution and plasticity. Nat Rev Genet. 2012;13: 163–174. 10.1038/nrg3161 22310892 PMC3595575

[10] Bopp D, Saccone G, Beye M. Sex determination in insects: variations on a common theme. Sex Dev. 2013;8: 20–28. 10.1159/000356458 24335049

[11] Dubendorfer A, Hediger M, Burghardt G, Bopp D. *Musca domestica*, a window on the evolution of sex-determining mechanisms in insects. Int J Dev Biol. 2002;46: 75–79. 11902690

[12] Luo SD, Shi GW, Baker BS. Direct targets of the *D*. *melanogaster* DSXF protein and the evolution of sexual development. Development. 2011;138: 2761–2771. 10.1242/dev.065227 21652649 PMC3109601

[13] Leimar O, Hartfelder K, Laubichler MD, Page RE Jr. Development and evolution of caste dimorphism in honeybees—a modeling approach. Ecol Evol. 2012;2: 3098–3109. 10.1002/ece3.414 23301175 PMC3539003

[14] Johnson BR, Borowiec ML, Chiu JC, Lee EK, Atallah J, Ward PS. Phylogenomics resolves evolutionary relationships among ants, bees, and wasps. Current Biology. 2013;23: 2058–2062. 10.1016/j.cub.2013.08.050 24094856

[15] Simola DF, Wissler L, Donahue G, Waterhouse RM, Helmkampf M, Roux J, et al Social insect genomes exhibit dramatic evolution in gene composition and regulation while preserving regulatory features linked to sociality. Genome Research. 2013;23: 1235–1247. 10.1101/gr.155408.113 23636946 PMC3730098

[16] Kapheim KM, Pan H, Li C, Salzberg SL, Puiu D, Magoc T, et al Genomic signatures of evolutionary transitions from solitary to group living. Science. 2015;348: 1139–1143. 10.1126/science.aaa4788 25977371 PMC5471836

[17] Patalano S, Vlasova A, Wyatt C, Ewels P, Camara F, Ferreira PG, et al Molecular signatures of plastic phenotypes in two eusocial insect species with simple societies. Proc Natl Acad Sci U S A. 2015. 10.1073/pnas.1515937112 PMC4653166 26483466

[18] Schrempf A, Aron S, Heinze J. Sex determination and inbreeding depression in an ant with regular sib-mating. Heredity. 2006;97: 75–80. 16705320 10.1038/sj.hdy.6800846

[19] Schrader L, Kim JW, Ence D, Zimin A, Klein A, Wyschetzki K, et al Transposable element islands facilitate adaptation to novel environments in an invasive species. Nature Communications. 2014;5: 5495 10.1038/ncomms6495 25510865 PMC4284661

[20] Oliveira DCSG, Werren JH, Verhulst EC, Giebel JD, Kamping A, Beukeboom LW, et al Identification and characterization of the doublesex gene of Nasonia. Insect Molecular Biology. 2009;18: 315–324. 10.1111/j.1365-2583.2009.00874.x 19523063 PMC2872477

[21] Cho S, Huang ZY, Zhang J. Sex-Specific Splicing of the Honeybee doublesex Gene Reveals 300 Million Years of Evolution at the Bottom of the Insect Sex-Determination Pathway. Genetics. 2007;177: 1733–1741. 17947419 10.1534/genetics.107.078980 PMC2147941

[22] Nipitwattanaphon M, Wang J, Ross KG, Riba-Grognuz O, Wurm Y, Khurewathanakul C, et al Effects of ploidy and sex-locus genotype on gene expression patterns in the fire ant Solenopsis invicta. Proc. Royal Soc B. 2014;281. 10.1098/rspb.2014.1776 PMC4240987 25355475

[23] Loehlin DW, Oliveira DCSG, Edwards R, Giebel JD, Clark ME, Cattani MV, et al Non-coding changes cause sex-specific wing size differences between closely related species of *Nasonia*. PLoS Genet. 2009;6: e1000821–e1000821. 10.1371/journal.pgen.1000821 PMC2799512 20090834

[24] Kunte K, Zhang W, Tenger-Trolander A, Palmer DH, Martin A, Reed RD, et al doublesex is a mimicry supergene. Nature. 2014;: 1–14. 10.1038/nature13112 24598547

[25] Eirín-López JM, Sánchez L. The comparative study of five sex-determining proteins across insects unveils high rates of evolution at basal components of the sex determination cascade. Development Genes and Evolution. 2015;225: 23–30. 10.1007/s00427-015-0491-6 25613749

[26] Kamping A, Katju V, Beukeboom LW, Werren JH. Inheritance of gynandromorphism in the parasitic wasp *Nasonia vitripennis*. Genetics. 2007;175: 1321–1333. 17179086 10.1534/genetics.106.067082 PMC1840083

[27] Michez D, Rasmont P, Terzo M, Vereecken NJ. A synthesis of gynandromorphy among wild bees (Hymenoptera: Apoidea), with an annotated description of several new cases. Annales de la Société Entomologique de France. 2009;45: 365–375.

[28] Yang AS, Abouheif E. Gynandromorphs as indicators of modularity and evolvability in ants. J Exp Zool (Mol Dev Evol). 2011;316: 313–318. 10.1002/jez.b.21407 21404423

[29] Heinze J, Trenkle S. Male polymorphism and gynandromorphs in the ant *Cardiocondyla emeryi*. Naturwissenschaften. 1997;84: 129–131.

[30] Yoshizawa J, Mimori K, Yamauchi K, Tsuchida K. Sex mosaics in a male dimorphic ant *Cardiocondyla kagutsuchi*. Naturwissenschaften. 2008;96: 49–55. 10.1007/s00114-008-0447-z 18797835

[31] Schrader L, Simola DF, Heinze J, Oettler J. Sphingolipids, transcription factors, and conserved toolkit genes: Developmental plasticity in the ant *Cardiocondyla obscurior*. Molecular Biology and Evolution. 2015;32: 1474–1486. 10.1093/molbev/msv039 25725431 PMC4615751

[32] Anders S, Reyes A, Huber W. Detecting differential usage of exons from RNA-seq data. Genome Research. 2012;22: 2008–2017. 10.1101/gr.133744.111 22722343 PMC3460195

[33] Alexa A, Rahnenführer J. topGO: Enrichment analysis for Gene Ontology. R package version. 2015;: 1–37.

[34] Luo SD, Baker BS. Constraints on the evolution of a doublesex target gene arising from doublesex's pleiotropic deployment. Proc Nat Acad Sci USA. 2015;112: E852–E861. 10.1073/pnas.1501192112 25675536 PMC4345571

[35] Tanaka K, Barmina O, Sanders LE, Arbeitman MN, Kopp A. Evolution of sex-specific traits through changes in HOX-dependent doublesex expression. Plos Biol. 2011;9: e1001131–e1001131. 10.1371/journal.pbio.1001131 21886483 PMC3160335

[36] Kijimoto T, Moczek AP. Diversification of doublesex function underlies morph-, sex-, and species-specific development of beetle horns. Proc Nat Acad Sci USA 2012. 10.1073/pnas.1118589109 PMC3528601 23184999

[37] Gotoh H, Miyakawa H, Ishikawa A, Ishikawa Y, Sugime Y, Emlen DJ, et al Developmental link between sex and nutrition; doublesex regulates sex-specific mandible growth via juvenile hormone signaling in stag beetles. PLoS Genet. 2013;10: e1004098–e1004098. 10.1371/journal.pgen.1004098 PMC3894178 24453990

[38] Emlen DJ, Warren IA, Johns A, Dworkin I, Lavine LC. A mechanism of extreme growth and reliable signaling in sexually selected ornaments and weapons. Science. 2012;337: 860–864. 10.1126/science.1224286 22837386

[39] Schrempf A, Heinze J. Proximate mechanisms of male morph determination in the ant *Cardiocondyla obscurior*. Evol Dev. 2006;8: 266–272. 16686637 10.1111/j.1525-142X.2006.00097.x

[40] Brito DV, Silva CGN, Hasselmann M, Viana LS, Astolfi-Filho S, Carvalho-Zilse GA. Molecular characterization of the gene feminizer in the stingless bee *Melipona interrupta* (Hymenoptera: Apidae) reveals association to sex and caste development. Insect Biochemistry and Molecular Biology. 2015;66: 24–30. 10.1016/j.ibmb.2015.09.008 26393998

[41] Hasselmann M, Lechner S, Schulte C, Beye M. Origin of a function by tandem gene duplication limits the evolutionary capability of its sister copy. Proc Nat Acad Sci USA. 2010;107: 13378–13383. 10.1073/pnas.1005617107 20624976 PMC2922136

[42] Koch V, Nissen I, Schmitt BD, Beye M. Independent evolutionary origin of *fem* paralogous genes and complementary sex determination in hymenopteran insects. PLoS ONE. 2014;9: e91883–11. 10.1371/journal.pone.0091883 24743790 PMC3990544

[43] Benevolenskaya EV, Frolov MV, Birchler JA. *Krüppel homolog* (*Kr h*) is a dosage-dependent modifier of gene expression in *Drosophila*. Genet Res. 2000;75: 137–142. 10816971 10.1017/s0016672399004437

[44] Minakuchi C, Zhou X, Riddiford LM. *Kruppel homolog 1* (*Kr-h1*) mediates juvenile hormone action during metamorphosis of *Drosophila melanogaster*. Mech Dev. 2008;125: 91–105. 18036785 10.1016/j.mod.2007.10.002 PMC2276646

[45] Minakuchi C, Namiki T, Shinoda T. *Krüppel homolog 1*, an early juvenile hormone-response gene downstream of *Methoprene-tolerant*, mediates its anti-metamorphic action in the red flour beetle *Tribolium castaneum*. Dev Biol. 2009;325: 341–350. 10.1016/j.ydbio.2008.10.016 19013451

[46] West-Eberhard MJ. Flexible strategy and social evolution Animal Societies, Theories and Facts. Tokyo: Japan Scientific Societies Press; 1987.

[47] Linksvayer TA, Wade MJ. The evolutionary origin and elaboration of sociality in the aculeate hymenoptera: Maternal effects, sib-social effects, and heterochrony. Q Rev Biol. 2005;80: 317–336. 16250466 10.1086/432266

[48] Kamakura M. Royalactin induces queen differentiation in honeybees. Nature. 2011;473: 478–483. 10.1038/nature10093 21516106

[49] Wang Y, Amdam GV, Rueppell O, Wallrichs MA, Fondrk MK, Kaftanoglu O, et al *PDK1* and *HR46* gene homologs tie social behavior to ovary signals. PLoS ONE. 2008;4: e4899–e4899. 10.1371/journal.pone.0004899 PMC2659776 19340296

[50] Mutti NS, Dolezal AG, Wolschin F, Mutti JS, Gill KS, Amdam GV. IRS and TOR nutrient-signaling pathways act via juvenile hormone to influence honey bee caste fate. Journal of Experimental Biology. 2011;214: 3977–3984. 10.1242/jeb.061499 22071189 PMC3212421

[51] Wheeler DE, Frederik Nijhout H. Soldier determination in *Pheidole bicarinata*: Effect of methoprene on caste and size within castes. J Insect Physiol. 1983;29: 847–854.

[52] Graham AM, Munday MD, Kaftanoglu O, Page RE, Amdam GV, Rueppell O. Support for the reproductive ground plan hypothesis of social evolution and major QTL for ovary traits of Africanized worker honey bees (*Apis mellifera* L.). BMC Evol Biol. 2011;11: 95 10.1186/1471-2148-11-95 21489230 PMC3100260

[53] Kucharski R, Maleszka J, Foret S, Maleszka R. Nutritional control of reproductive status in honeybees via DNA methylation. Science. 2008;319: 1827–1830. 10.1126/science.1153069 18339900

[54] Li Q, Wang Z, Lian J, Schiøtt M, Jin L, Zhang P, et al Caste-specific RNA editomes in the leaf-cutting ant *Acromyrmex echinatior*. Nature Communications. 2013;5: 4943–4943. 10.1038/ncomms5943 PMC4200514 25266559

[55] Simola DF, Ye C, Mutti NS, Dolezal K, Bonasio R, Liebig J, et al A chromatin link to caste identity in the carpenter ant *Camponotus floridanus*. Genome Research. 2013;23: 486–496. 10.1101/gr.148361.112 23212948 PMC3589537

[56] Edgar RC. MUSCLE: multiple sequence alignment with high accuracy and high throughput. Nucl Acids Res. 2004;32: 1792–1797. 15034147 10.1093/nar/gkh340 PMC390337

[57] Hall BG. Building phylogenetic trees from molecular data with MEGA. Molecular Biology and Evolution. 2013;30: 1229–1235. 10.1093/molbev/mst012 23486614

[58] Love MI, Huber W, Anders S. Moderated estimation of fold change and dispersion for RNA-Seq data with DESeq2. bioRxiv. 2014 Available: http://biorxiv.org/content/biorxiv/early/2014/02/19/002832.full.pdf 10.1186/s13059-014-0550-8 PMC4302049 25516281

[59] Livak KJ, Schmittgen TD. Analysis of Relative Gene Expression Data Using Real-Time Quantitative PCR and the 2−ΔΔCT Method. Methods. 2001;25: 402–408. 11846609 10.1006/meth.2001.1262

[60] Dobin A, Davis CA, Schlesinger F, Drenkow J, Zaleski C, Jha S, et al STAR: ultrafast universal RNA-seq aligner. Bioinformatics. 2013;29: 15–21. 10.1093/bioinformatics/bts635 23104886 PMC3530905

[61] Schmieder S, Colinet D, eacute MENP. Tracing back the nascence of a new sex-determination pathway to the ancestor of bees and ants. Nature Communications. Nature Publishing Group; 2012;3: 895–7. 10.1038/ncomms1898 PMC3621418 22692538

[62] Privman E, Wurm Y, Keller L. Duplication and concerted evolution in a master sex determiner under balancing selection. Proc Biol Sci. 2013;280: 20122968–20122968. 10.1098/rspb.2012.2968 23466984 PMC3619454

[63] Murtagh F, Legendre P. Ward’s hierarchical agglomerative clustering method: Which algorithms implement Ward’s criterion? J Classif. 2014;31: 274–295.

