# Supplementary material for: Evolution of Social Insect Polyphenism Facilitated by the Sex Differentiation Cascade
Source: PLoS Genet. 2016 Mar 31;12(3):e1005952. doi: 10.1371/journal.pgen.1005952 (PMC4816456; doi:10.1371/journal.pgen.1005952)
Supplement: S1 Table — For each family, a Japanese (JP) Cardiocondyla obscurior queen was mated with a Brazilian (BR) male. Emerging F1 individuals were genotyped using three population-specific microsatellite markers. This showed that all F1 males (EM = ergatoid, wingless males, WM = winged males) and one gynandromorph (GY) exclusively carried the maternal (JP) allele, whereas emerging females (QU = queens, WO = workers) carried both parental alleles (JP+BR). Sample sizes are given in parenthesis. (DOCX) [file pgen.1005952.s001.docx]

**S1 Table**

| family | F1-EM | F1-WM | F1-QU | F1-WO | F1-GY |
| --- | --- | --- | --- | --- | --- |
| sd5 | JP (4) | - | JP+BR (5) | JP+BR (4) | - |
| sd9 | JP (4) | JP (1) | JP+BR (4) | JP+BR (4) | JP (1) |
| sd11 | JP (4) | - | JP+BR (4) | JP+BR (4) | - |
| sd12 | JP (5) | JP (2) | JP+BR (4) | JP+BR (4) | - |
| sd18 | JP (6) | - | JP+BR (5) | JP+BR (6) | - |
| sum | 23 | 3 | 22 | 22 | 1 |
