# Supplementary material for: Evolution of Social Insect Polyphenism Facilitated by the Sex Differentiation Cascade
Source: PLoS Genet. 2016 Mar 31;12(3):e1005952. doi: 10.1371/journal.pgen.1005952 (PMC4816456; doi:10.1371/journal.pgen.1005952)
Supplement: S2 Table — We used previously published RNAseq data and analyzed expression in females vs. males with DESeq2. Only Cobs_01393 is differentially expressed between the sexes. (DOCX) [file pgen.1005952.s002.docx]

**S2 Table**

| transcript | base Mean | log2FoldChange | padj |
| --- | --- | --- | --- |
| Cobs_01393 | 53.22 | 1.74 | < 0.001 |
| Cobs_07724 | 17.83 | 0.09 | 0.83 |
| Cobs_09254 | 11.49 | -0.11 | 0.81 |
| Cobs_18158 | 46.63 | 0.04 | 0.90 |
