# Supplementary material for: Evolution of Social Insect Polyphenism Facilitated by the Sex Differentiation Cascade
Source: PLoS Genet. 2016 Mar 31;12(3):e1005952. doi: 10.1371/journal.pgen.1005952 (PMC4816456; doi:10.1371/journal.pgen.1005952)
Supplement: S3 Table — Positions are based on genome version Cobs1.4. (DOCX) [file pgen.1005952.s003.docx]

**S3 Table**

| exon | positions on scf0005 | size in bp |
| --- | --- | --- |
| 1 | 3,962,553 – 3,962,614 | 62 |
| 2 | 3,962,850 – 3,963,207 | 358 |
| 3 | 3,963,485 – 3,963,524 | 40 |
| 4 | 3,973,929 – 3,974,117 | 189 |
| 5 (female-specific) | 3,974,295 – 3,975,124 | 830 |
| 6 (male-specific) | 3,989,920 – 3,990,340 | 421 |
| 7 (male-specific) | 3,999,828 – 4,004,104 | 4277 |
