# Supplementary material for: Evolution of Social Insect Polyphenism Facilitated by the Sex Differentiation Cascade
Source: PLoS Genet. 2016 Mar 31;12(3):e1005952. doi: 10.1371/journal.pgen.1005952 (PMC4816456; doi:10.1371/journal.pgen.1005952)
Supplement: S4 Table — Sex mosaics are classified as ergatandromorph (E, intersex worker (WO) / ergatoid male (EM)) or gynandromorph (G, intersex queen (QU) / winged male (WM)). Descriptions mainly focus on head morphology, in which differences are most prominent. (DOCX) [file pgen.1005952.s004.docx]

**S4 Table**

| id | G/E | life stage | morphological description |
| --- | --- | --- | --- |
| # 4* | G | pupa | laterally separated in QU and WM |
| # 6* | G | pupa | left half: QU + male antennae, right half: WM + QU antennae |
| # 8* | E | pupa | left half: EM, right half: WO |
| # 10* | G | pupa | left half: WM, right half: QU |
| # 7 | G | adult | laterally separated in QU and WM |
| # 9 | E | adult | laterally separated in WO and EM |
| # 11 | G | adult | laterally separated in QU and WM |
| # 12 | G | adult | WM-like big eyes; both antennae QU-like with short scapus |
| # 14 | G | adult | WM-like big eyes; right antennae QU-like with short scapus, left antennae WM-like with long scapus |
| # 15 | G | adult | WM-like big eyes; right antenna WM-like with long scapus, left antenna QU-like with short scapus |
| # 16 | G | adult | laterally separated in QU and WM |
| # 17 | E | pupa | eyes WO-like, EM-like saber-shaped mandibles |
| # 18 | G | adult | WM-like big eyes, both antennae QU-like with short scapus |
| # 19 | G | adult | WM head, QU thorax (dealate) |
| # 26 | G | adult | laterally separated in QU and WM |
| # 27 | G | adult | laterally separated in QU and WM |
| # 34 | E | pupa | laterally separated in WO and EM |
| * = used in qPCR | | | |
