# Supplementary material for: Evolution of Social Insect Polyphenism Facilitated by the Sex Differentiation Cascade
Source: PLoS Genet. 2016 Mar 31;12(3):e1005952. doi: 10.1371/journal.pgen.1005952 (PMC4816456; doi:10.1371/journal.pgen.1005952)
Supplement: S5 Table — RNAseq data was used to generate per exon count tables for the corrected dsx gene model for 3rd instar larvae which were analyzed with Kruskal-Wallis rank sum tests and pairwise Wilcoxon-Test with Benjamini-Hochberg correction. (DOCX) [file pgen.1005952.s005.docx]

**S5 Table**

| exon | kruskal test across all morphs | pairwise wilcox test | | | | | |
| --- | --- | --- | --- | --- | --- | --- | --- |
|  |  | EM-WM | EM-WO | EM- QU | WM-WO | WM-QU | WO-QU |
| exon1 | X^2^=16.13, df=3, p=0.011 | 0.0390 | 0.0150 | 0.0150 | 0.0640 | 0.0210 | 0.2500 |
| exon2 | X^2^=22.71, df=3, p<0.001 | 0.0035 | 0.0012 | 0.0012 | 0.0049 | 0.0012 | 0.0530 |
| exon3 | X^2^=21.68, df=3, p<0.001 | 0.0031 | 0.0031 | 0.0031 | 0.2086 | 0.0031 | 0.0123 |
| exon4 | X^2^=19.06, df=3, p<0.001 | 0.0061 | 0.0047 | 0.0017 | 0.1168 | 0.0017 | 0.3176 |
| exon5 | X^2^=16.49, df=3, p<0.001 | 0.0568 | 0.0524 | 0.6200 | 0.0017 | 0.0017 | 0.0636 |
| exon6 | X^2^=22.70, df=3, p<0.001 | 0.0026 | 0.0026 | 0.0026 | 0.0026 | 0.0026 | 0.7961 |
| exon7 | X^2^=24.10, df=3, p<0.001 | 0.0007 | 0.0007 | 0.0007 | 0.0007 | 0.0007 | 0.0262 |
