# Supplementary material for: Evolution of Social Insect Polyphenism Facilitated by the Sex Differentiation Cascade
Source: PLoS Genet. 2016 Mar 31;12(3):e1005952. doi: 10.1371/journal.pgen.1005952 (PMC4816456; doi:10.1371/journal.pgen.1005952)
Supplement: S6 Table — Bold numbers indicate loadings that fell in the 10% or 90% quantiles for PC1, PC2 & PC4. The 179 sex-biased exons were extracted with DEXseq using a false discovery rate of 0.005. (DOCX) [file pgen.1005952.s006.docx]

**S6 Table**

| Exon ID | PC1 | PC2 | PC3 | PC4 |
| --- | --- | --- | --- | --- |
| Cobs_00321.mRNA.1.E002 | 0.0562382873 | -0.0469522101 | -0.0522020161 | -0.0248998208 |
| Cobs_00321.mRNA.1.E006 | 0.0209059028 | -0.0535784210 | -0.0663160451 | -0.0033977391 |
| Cobs_00374.mRNA.1.E005 | **0.0988034890** | 0.0781461284 | 0.0241897146 | 0.0186604289 |
| Cobs_00374.mRNA.1.E007 | 0.0776478991 | 0.0879914611 | 0.0077047346 | 0.0538194057 |
| Cobs_00374.mRNA.1.E008 | 0.0777561199 | 0.0063557308 | -0.0490479993 | 0.0042016531 |
| Cobs_01020.mRNA.1.E002 | 0.0570320853 | **-0.1371031360** | -0.0289168756 | 0.0582219468 |
| Cobs_01154.mRNA.1.E008 | 0.0259214593 | 0.0978799515 | 0.0871926940 | **0.0875834806** |
| Cobs_01393.mRNA.1.E005 | 0.0752227118 | -0.0272423911 | -0.0863315256 | **0.1199763586** |
| Cobs_01393.mRNA.1.E006 | -0.0983936018 | 0.0270897173 | -0.0421485645 | **0.1370003723** |
| Cobs_01393.mRNA.1.E007 | **-0.1026979831** | 0.0263004114 | -0.0524285714 | **0.1306402712** |
| Cobs_01404.mRNA.1.E007 | -0.0735259289 | 0.0610078176 | 0.0554938947 | **-0.1422011784** |
| Cobs_01404.mRNA.1.E014 | 0.0517963573 | **0.1515488442** | 0.0732645364 | -0.0218614922 |
| Cobs_01504.mRNA.1.E002 | 0.0016959771 | **0.1402418831** | -0.0135906012 | -0.0855509310 |
| Cobs_01907.mRNA.1.E011 | -0.0891935029 | -0.0946207759 | -0.0025935604 | -0.0439723528 |
| Cobs_02962.mRNA.1.E015 | -0.0006626646 | **-0.1499880879** | -0.0123599697 | -0.0860383784 |
| Cobs_03219.mRNA.1.E001 | -0.0770700977 | 0.0872844848 | -0.0793515483 | -0.0085188897 |
| Cobs_03241.mRNA.1.E007 | -0.0895224483 | 0.0348534133 | 0.0660123650 | 0.0117203295 |
| Cobs_03321.mRNA.1.E001 | 0.0627406550 | **-0.1492376292** | -0.0363229466 | -0.0658655640 |
| Cobs_03321.mRNA.1.E002 | 0.0704399383 | **-0.1496325800** | -0.0357914249 | -0.0260744246 |
| Cobs_03321.mRNA.1.E004 | **0.0936470138** | **-0.1123395150** | -0.0604334007 | 0.0209392194 |
| Cobs_03321.mRNA.1.E005 | **0.0928920791** | **-0.1175679389** | -0.0504467044 | 0.0350537294 |
| Cobs_03549.mRNA.1.E001 | 0.0872420519 | -0.0564780017 | -0.0214028332 | -0.0448670244 |
| Cobs_03876.mRNA.1.E007 | **-0.1057160446** | 0.0006825815 | -0.0217198809 | -0.0939389563 |
| Cobs_04124.mRNA.1.E003 | 0.0056562913 | 0.0811835272 | -0.1426600756 | -0.0226364374 |
| Cobs_04205.mRNA.1.E008 | 0.0503024972 | -0.0879869261 | 0.0214201339 | 0.0774399590 |
| Cobs_04247.mRNA.1.E001 | -0.0059591657 | -0.0225328679 | -0.0144477939 | -0.0164814469 |
| Cobs_04247.mRNA.1.E004 | -0.0865993406 | -0.0286615794 | 0.1045264707 | -0.0109143738 |
| Cobs_04247.mRNA.1.E005 | -0.0912547440 | -0.0345202184 | 0.1076812888 | -0.0632772076 |
| Cobs_04249.mRNA.1.E001 | 0.0356037902 | **0.1351604504** | 0.1222119190 | -0.0336030054 |
| Cobs_04249.mRNA.1.E002 | 0.0395380636 | **0.1377974774** | 0.1202601310 | -0.0374530368 |
| Cobs_04249.mRNA.1.E004 | -0.0939417760 | 0.0227188679 | -0.1104284859 | -0.0692015158 |
| Cobs_04339.mRNA.1.E002 | -0.0253937630 | 0.0424600334 | -0.0594897474 | **0.1833834904** |
| Cobs_04342.mRNA.1.E004 | -0.0920855018 | -0.0589812343 | -0.0787523569 | -0.0330124227 |
| Cobs_04587.mRNA.1.E004 | 0.0629235699 | 0.0528072046 | -0.1639305528 | -0.0318304980 |
| Cobs_04652.mRNA.1.E008 | **0.1000144284** | 0.0743249095 | 0.0280407205 | -0.0004933052 |
| Cobs_04840.mRNA.1.E012 | **-0.1097961436** | -0.0511257274 | -0.0437184486 | -0.0199934947 |
| Cobs_04840.mRNA.1.E013 | **-0.1054513384** | 0.0279910100 | 0.0238100727 | -0.0962623918 |
| Cobs_04840.mRNA.1.E014 | -0.0992775999 | -0.0090532787 | -0.0118330020 | -0.0554953936 |
| Cobs_04840.mRNA.1.E018 | -0.0997497449 | 0.0125108153 | -0.0648554888 | -0.0654761828 |
| Cobs_04840.mRNA.1.E019 | -0.0857149882 | -0.0390511265 | -0.0581807849 | **-0.1266666118** |
| Cobs_04840.mRNA.1.E020 | -0.0931041770 | -0.0416563358 | -0.0503802388 | **-0.1142545550** |
| Cobs_04840.mRNA.1.E027 | 0.0663718368 | **-0.1121115144** | -0.0051625831 | -0.0061544746 |
| Cobs_04954.mRNA.1.E004 | 0.0659148888 | **0.1215326744** | 0.0975162350 | 0.0078514808 |
| Cobs_04954.mRNA.1.E007 | 0.0338963657 | **0.1343001572** | 0.1386968379 | -0.0376432495 |
| Cobs_05429.mRNA.1.E021 | 0.0335194626 | **-0.1107957920** | 0.0866388582 | 0.0742242668 |
| Cobs_05728.mRNA.1.E012 | 0.0849394796 | -0.0743109246 | -0.0891492315 | **-0.1217624595** |
| Cobs_05728.mRNA.1.E013 | 0.0811590157 | -0.0689587947 | -0.0900994456 | **-0.1344718007** |
| Cobs_05728.mRNA.1.E015 | **0.1053835877** | -0.0440799648 | -0.0861253515 | -0.0953617875 |
| Cobs_05728.mRNA.1.E016 | **0.1092376107** | -0.0395369286 | -0.0697426526 | -0.0861952490 |
| Cobs_05822.mRNA.1.E004 | 0.0710459452 | **-0.1250571023** | -0.0493063265 | 0.0370597079 |
| Cobs_05838.mRNA.1.E017 | **0.0930996779** | -0.0085289707 | -0.1483049666 | -0.0405078520 |
| Cobs_05898.mRNA.1.E002 | 0.0684530125 | 0.0883671166 | 0.0837715341 | -0.0607667855 |
| Cobs_06565.mRNA.1.E001 | 0.0417674481 | **-0.1102425688** | -0.1090104584 | -0.0449167273 |
| Cobs_06593.mRNA.1.E003 | **-0.1048160252** | 0.0123267175 | -0.0195574944 | 0.1054393843 |
| Cobs_06933.mRNA.1.E005 | 0.0764959126 | 0.0451070667 | -0.1243731488 | -0.0035664523 |
| Cobs_07060.mRNA.1.E005 | 0.0562128432 | **-0.1098553340** | -0.0327398337 | -0.0967354323 |
| Cobs_07060.mRNA.1.E007 | 0.0696803503 | -0.0796269641 | -0.0691106431 | -0.0856100089 |
| Cobs_07270.mRNA.1.E001 | 0.0176370090 | 0.0050354935 | 0.0411620002 | -0.0481000547 |
| Cobs_07270.mRNA.1.E002 | 0.0036710805 | 0.0282291310 | 0.0886117710 | 0.0695161889 |
| Cobs_07270.mRNA.1.E006 | -0.0317105271 | 0.0141466398 | 0.0663072679 | -0.0086866251 |
| Cobs_07270.mRNA.1.E007 | -0.0193510669 | 0.0176931113 | 0.0353681526 | 0.0566708853 |
| Cobs_07270.mRNA.1.E008 | 0.0247357353 | 0.0526113501 | -0.0395991067 | 0.0551707820 |
| Cobs_07270.mRNA.1.E009 | -0.0792805534 | -0.0584768615 | 0.0795268584 | -0.1037892954 |
| Cobs_07270.mRNA.1.E010 | -0.0831577975 | -0.0588943538 | 0.0798053896 | -0.0700194650 |
| Cobs_07270.mRNA.1.E011 | -0.0925733224 | -0.0403000965 | 0.0965028452 | -0.0601201052 |
| Cobs_07270.mRNA.1.E014 | -0.0811809833 | -0.0569213253 | 0.0640064847 | **-0.1500016866** |
| Cobs_07270.mRNA.1.E015 | -0.0728829201 | -0.0582242860 | 0.0822015410 | **-0.1350003875** |
| Cobs_07270.mRNA.1.E016 | -0.0818668733 | -0.0603957907 | 0.0900087708 | -0.0916281460 |
| Cobs_07560.mRNA.1.E001 | -0.0962700875 | 0.0290439890 | -0.0587213684 | 0.0393211818 |
| Cobs_07560.mRNA.1.E003 | 0.0921253848 | -0.0182648789 | -0.1032532346 | -0.0479360658 |
| Cobs_07560.mRNA.1.E004 | 0.0797172627 | -0.0002137049 | -0.1114493102 | -0.0982133335 |
| Cobs_07606.mRNA.1.E020 | **-0.1118297296** | 0.0024240695 | -0.1107470635 | 0.0300095574 |
| Cobs_07682.mRNA.1.E010 | 0.0582640932 | 0.0802506622 | 0.0488494809 | **-0.1094219505** |
| Cobs_07682.mRNA.1.E011 | 0.0713310860 | 0.0815219093 | 0.0609168669 | **-0.1094844429** |
| Cobs_07682.mRNA.1.E012 | 0.0602911509 | 0.0854491656 | 0.0621900794 | **-0.1270765090** |
| Cobs_07682.mRNA.1.E021 | -0.0560209098 | 0.0724286551 | 0.1317773037 | **-0.1127351340** |
| Cobs_08315.mRNA.1.E018 | -0.0459039203 | -0.0688403733 | 0.0704164252 | **-0.1415938024** |
| Cobs_08519.mRNA.1.E005 | 0.0887047414 | -0.0422317974 | -0.1292681106 | -0.0404120195 |
| Cobs_08680.mRNA.1.E005 | -0.0687866380 | 0.0051932459 | -0.0363867875 | -0.0125091250 |
| Cobs_08682.mRNA.1.E001 | 0.0733209189 | **-0.1216389725** | -0.0174783357 | -0.0009056975 |
| Cobs_08682.mRNA.1.E003 | **-0.1022600015** | 0.0111618175 | -0.0808747737 | **0.1139248490** |
| Cobs_08765.mRNA.1.E001 | 0.0616058993 | -0.0576938929 | 0.0398078055 | 0.0051611451 |
| Cobs_09050.mRNA.1.E004 | 0.0674403708 | 0.0630585960 | -0.0138253133 | -0.1076868685 |
| Cobs_09050.mRNA.1.E007 | -0.0362849706 | 0.0952325307 | -0.1017677965 | **-0.1250339366** |
| Cobs_09050.mRNA.1.E008 | -0.0441826812 | 0.0888968170 | -0.0895999365 | **-0.1400029457** |
| Cobs_09050.mRNA.1.E009 | -0.0324334885 | 0.0985614853 | -0.1073047609 | **-0.1550594271** |
| Cobs_09264.mRNA.1.E008 | 0.0584143988 | 0.0620081748 | -0.0202413285 | 0.0473076855 |
| Cobs_09331.mRNA.1.E006 | 0.0691173809 | -0.0530090865 | -0.0635604435 | 0.0239948263 |
| Cobs_09342.mRNA.1.E008 | **0.0979050052** | 0.0709239011 | -0.0781798985 | -0.0288999326 |
| Cobs_09658.mRNA.1.E003 | -0.0676272380 | 0.0777055469 | -0.0129724379 | -0.0018916338 |
| Cobs_09658.mRNA.1.E008 | -0.0425239715 | 0.0781639748 | -0.1407176502 | -0.0241373541 |
| Cobs_09658.mRNA.1.E020 | **0.1036047503** | -0.0378552027 | -0.1210111711 | -0.0251442178 |
| Cobs_09658.mRNA.1.E021 | **0.1068589058** | -0.0436906731 | -0.1112571209 | -0.0301942771 |
| Cobs_09658.mRNA.1.E022 | **0.1061528794** | -0.0348364113 | -0.1149623731 | -0.0487857966 |
| Cobs_09895.mRNA.1.E012 | -0.0686270412 | 0.0831334236 | -0.0736872057 | -0.0349327826 |
| Cobs_10135.mRNA.1.E001 | -0.0422443780 | -0.0438499936 | -0.0607409909 | **-0.1723455107** |
| Cobs_10135.mRNA.1.E002 | 0.0432025775 | -0.0567729370 | -0.1054888378 | -0.0774635115 |
| Cobs_10155.mRNA.1.E001 | -0.1006603208 | -0.0146498091 | -0.0298257150 | 0.0584648543 |
| Cobs_10249.mRNA.1.E005 | -0.0508352634 | 0.0555190408 | 0.0142071490 | -0.0655331308 |
| Cobs_10318.mRNA.1.E001 | -0.0713221665 | 0.0918495331 | 0.0364389756 | -0.0863922601 |
| Cobs_10679.mRNA.1.E007 | **0.0990014265** | -0.0040951762 | -0.0321325993 | -0.0338906578 |
| Cobs_10686.mRNA.1.E004 | -0.0951010503 | 0.0240402354 | -0.0698104309 | **0.1239063399** |
| Cobs_10755.mRNA.1.E004 | 0.0669928780 | 0.0570598320 | -0.0463348160 | **0.1202727480** |
| Cobs_10939.mRNA.1.E011 | -0.0429223561 | -0.0054908365 | 0.1093785715 | -0.0618795340 |
| Cobs_11061.mRNA.1.E003 | -0.0090875798 | **-0.1314355719** | 0.0812395202 | **0.0913591000** |
| Cobs_11146.mRNA.1.E011 | 0.0690400597 | **0.1051375547** | 0.0209936049 | -0.0196831326 |
| Cobs_11539.mRNA.1.E001 | 0.0715162494 | **0.1023505479** | 0.0284929727 | **0.1083570685** |
| Cobs_11539.mRNA.1.E002 | -0.0640259976 | -0.0099304180 | -0.0329083163 | **0.1524632964** |
| Cobs_11673.mRNA.1.E003 | -0.0480845499 | -0.0046769280 | -0.0400688482 | -0.0729317711 |
| Cobs_11854.mRNA.1.E002 | 0.0667928997 | **-0.1136061746** | -0.0063148972 | -0.0478166717 |
| Cobs_12024.mRNA.1.E003 | **0.0973610344** | 0.0097371701 | -0.0752641859 | -0.0739590929 |
| Cobs_12024.mRNA.1.E006 | -0.0656009304 | **0.1275013470** | -0.0900714800 | -0.0288808270 |
| Cobs_12024.mRNA.1.E009 | -0.0740325530 | **0.0998158800** | -0.1125229548 | -0.0397625456 |
| Cobs_12024.mRNA.1.E010 | -0.0616677625 | **0.1307255933** | -0.0792081979 | -0.0356211696 |
| Cobs_12024.mRNA.1.E012 | -0.0652069616 | **0.1315692975** | -0.0713111765 | -0.0256511763 |
| Cobs_12024.mRNA.1.E014 | -0.0653527769 | **0.1076954742** | -0.0998658606 | -0.0167308590 |
| Cobs_12269.mRNA.1.E001 | 0.0762840000 | 0.0855582439 | -0.0542601085 | -0.0514465016 |
| Cobs_12269.mRNA.1.E004 | -0.0726983968 | **0.1002327288** | -0.1006608206 | 0.0637172228 |
| Cobs_12269.mRNA.1.E005 | -0.0759348103 | 0.0770418683 | -0.0588225035 | 0.0147178905 |
| Cobs_12269.mRNA.1.E006 | -0.0760003143 | 0.0970345300 | -0.0792093452 | 0.0848421752 |
| Cobs_12296.mRNA.1.E003 | **-0.1116698382** | 0.0057954363 | -0.0641288785 | -0.0337302535 |
| Cobs_12296.mRNA.1.E004 | **-0.1077903141** | -0.0167899405 | -0.1095983859 | 0.0135853723 |
| Cobs_12296.mRNA.1.E006 | **-0.1048772824** | 0.0059773416 | -0.0669533787 | 0.0733045471 |
| Cobs_12296.mRNA.1.E010 | 0.0426252350 | -0.0560399482 | 0.0277342049 | **0.1568083507** |
| Cobs_12296.mRNA.1.E011 | 0.0566102870 | -0.0607572525 | 0.0496290344 | **0.1574800578** |
| Cobs_12296.mRNA.1.E012 | 0.0376749395 | -0.0670643660 | -0.0106826181 | **0.1304610266** |
| Cobs_12302.mRNA.1.E014 | 0.0403836525 | -0.0138352663 | 0.0028241888 | -0.0119398657 |
| Cobs_12359.mRNA.1.E001 | 0.0195337865 | 0.0708711805 | 0.1233542800 | -0.0080550379 |
| Cobs_12359.mRNA.1.E011 | 0.0580935484 | 0.0836772361 | 0.1151622522 | -0.0340260625 |
| Cobs_12359.mRNA.1.E013 | 0.0824857727 | 0.0670909829 | 0.0198038890 | 0.0166588643 |
| Cobs_12359.mRNA.1.E018 | **-0.1062291699** | -0.0534953766 | -0.0225090992 | -0.0348680520 |
| Cobs_12359.mRNA.1.E020 | -0.0977898959 | -0.0483641059 | 0.0214106886 | -0.0257368707 |
| Cobs_12359.mRNA.1.E021 | **-0.1087821404** | -0.0327849447 | 0.0802310272 | 0.0090158166 |
| Cobs_12359.mRNA.1.E022 | **-0.1115455126** | -0.0367064469 | 0.0652272465 | 0.0301763646 |
| Cobs_12359.mRNA.1.E024 | **-0.1081852780** | -0.0139146636 | -0.0397573699 | -0.0698419724 |
| Cobs_12497.mRNA.1.E008 | -0.0909576464 | -0.0561074740 | 0.1202726163 | 0.0767377182 |
| Cobs_12519.mRNA.1.E002 | **0.0926462189** | 0.0165413616 | -0.0714085109 | -0.0804290907 |
| Cobs_12910.mRNA.1.E010 | 0.0414125749 | **0.1358258365** | 0.0718225411 | -0.0782091007 |
| Cobs_13971.mRNA.1.E006 | -0.0055882286 | -0.0283775684 | 0.0568499902 | 0.0503206621 |
| Cobs_14042.mRNA.1.E001 | **0.1079279128** | 0.0826460606 | 0.0235494851 | -0.0244945073 |
| Cobs_14042.mRNA.1.E003 | -0.0350497375 | **-0.1168862838** | -0.0273618692 | -0.0084846726 |
| Cobs_14042.mRNA.1.E004 | -0.0389630641 | **-0.1125577642** | 0.0189555622 | -0.0677494098 |
| Cobs_14726.mRNA.1.E001 | -0.0358643610 | 0.0771303818 | -0.1632061911 | 0.0528253378 |
| Cobs_14726.mRNA.1.E002 | -0.0465444829 | 0.0786262098 | -0.1410842356 | 0.0663365579 |
| Cobs_14726.mRNA.1.E003 | -0.0524339633 | 0.0609795740 | -0.1456727656 | 0.0573440049 |
| Cobs_14726.mRNA.1.E004 | -0.0230923960 | 0.0583610072 | -0.1239663597 | 0.0487567124 |
| Cobs_14726.mRNA.1.E005 | -0.0821946259 | 0.0744758323 | -0.1365683415 | 0.0136492522 |
| Cobs_14726.mRNA.1.E006 | **0.0998324945** | 0.0687706939 | -0.0232245446 | **0.0851115591** |
| Cobs_14732.mRNA.1.E002 | **0.0986371407** | 0.0059237625 | -0.0314611866 | 0.0476048891 |
| Cobs_14732.mRNA.1.E005 | -0.0940764916 | 0.0050943488 | -0.0978083118 | 0.0083284816 |
| Cobs_15127.mRNA.1.E001 | -0.0887239946 | -0.0443620782 | 0.0256253602 | -0.0026329274 |
| Cobs_15219.mRNA.1.E011 | 0.0128700122 | 0.0891599127 | 0.0497866967 | **-0.1349689379** |
| Cobs_15399.mRNA.1.E009 | -0.0878909571 | -0.0486980806 | 0.0815449116 | **0.0082752767** |
| Cobs_15543.mRNA.1.E003 | -0.0067243603 | -0.0766635768 | -0.0047438577 | -0.0014739423 |
| Cobs_15576.mRNA.1.E005 | 0.0425674173 | -0.0976250101 | -0.0085942852 | -0.0690836411 |
| Cobs_15576.mRNA.1.E006 | 0.0506267813 | -0.0893476115 | -0.0024390048 | -0.0673246445 |
| Cobs_15576.mRNA.1.E009 | 0.0923302600 | -0.0640820382 | -0.0280944917 | 0.0154278095 |
| Cobs_15676.mRNA.1.E011 | -0.0569739426 | **-0.1196721816** | -0.0208955217 | **-0.1170061469** |
| Cobs_15702.mRNA.1.E002 | **-0.1082999595** | -0.0393847267 | -0.0916959409 | 0.0727991730 |
| Cobs_15702.mRNA.1.E006 | **-0.1147517773** | -0.0230736623 | -0.0645811567 | 0.0604098802 |
| Cobs_15702.mRNA.1.E010 | 0.0283154402 | -0.0659191487 | -0.0635084916 | **0.1234883797** |
| Cobs_15807.mRNA.1.E013 | -0.0753479453 | -0.0381186652 | 0.0992682890 | -0.0221494212 |
| Cobs_15950.mRNA.1.E006 | 0.0508592781 | **0.1375654070** | 0.0513814211 | -0.0652501140 |
| Cobs_16311.mRNA.1.E005 | -0.0738887814 | 0.0108580241 | -0.0115083660 | -0.0228163669 |
| Cobs_16333.mRNA.1.E003 | -0.0419501532 | -0.0092349047 | -0.0747171140 | -0.0656606478 |
| Cobs_16333.mRNA.1.E004 | -0.0475158788 | -0.0117214955 | -0.0606550780 | -0.0621935787 |
| Cobs_16333.mRNA.1.E005 | -0.0389374725 | -0.0119939643 | -0.0632989341 | -0.0793198297 |
| Cobs_16333.mRNA.1.E006 | -0.0438658929 | -0.0159963561 | -0.0601022561 | -0.0670369262 |
| Cobs_16635.mRNA.1.E003 | 0.0654048947 | 0.0324086691 | -0.0606894379 | -0.0046155695 |
| Cobs_16641.mRNA.1.E003 | 0.0769809357 | 0.0456196250 | 0.0954157888 | 0.0460784660 |
| Cobs_17223.mRNA.1.E006 | -0.0250521090 | **-0.1364130330** | 0.0649810234 | -0.0756715369 |
| Cobs_17408.mRNA.1.E003 | 0.0900575275 | 0.0669644348 | 0.0340965154 | -0.0322146250 |
| Cobs_17408.mRNA.1.E004 | 0.0612223571 | **0.1108630300** | -0.0415525324 | -0.0822979169 |
| Cobs_17408.mRNA.1.E005 | 0.0566992016 | **0.1111408883** | -0.0006394764 | -0.0269494382 |
| Cobs_17408.mRNA.1.E007 | **-0.1235932661** | -0.0116278414 | -0.0542794849 | 0.0499657212 |
| Cobs_17408.mRNA.1.E010 | **-0.1123484872** | -0.0646543068 | -0.0628456217 | 0.0062875970 |
| Cobs_17852.mRNA.1.E005 | 0.0842531564 | 0.0077489791 | 0.0052408443 | **0.1328759123** |
| Cobs_17963.mRNA.1.E013 | **0.0968118245** | -0.0270663682 | -0.0302395941 | -0.0423850511 |
| Cobs_18218.mRNA.1.E010 | 0.0905119225 | 0.0967837180 | -0.0423275381 | 0.0385708616 |
