# Supplementary material for: Evolution of Social Insect Polyphenism Facilitated by the Sex Differentiation Cascade
Source: PLoS Genet. 2016 Mar 31;12(3):e1005952. doi: 10.1371/journal.pgen.1005952 (PMC4816456; doi:10.1371/journal.pgen.1005952)
Supplement: S7 Table — (DOCX) [file pgen.1005952.s007.docx]

**S7 Table**

| GO-ID | Term | Annotated | Significant | Expected | p-Value |
| --- | --- | --- | --- | --- | --- |
| GO:0036125 | fatty acid beta-oxidation multienzyme complex | 1 | 1 | 0 | 0.0015 |
| GO:0051301 | cell division | 176 | 3 | 0.19 | 0.0019 |
| GO:0016507 | mitochondrial fatty acid beta-oxidation multienzyme complex | 1 | 1 | 0 | 0.0061 |
| GO:0051642 | centrosome localization | 1 | 1 | 0 | 0.0119 |
| GO:0051383 | kinetochore organization | 3 | 1 | 0 | 0.0157 |
| GO:0030037 | actin filament reorganization involved in cell cycle | 2 | 1 | 0 | 0.0178 |
| GO:0048132 | female germ-line stem cell asymmetric division | 3 | 1 | 0 | 0.0191 |
| GO:0030723 | ovarian fusome organization | 5 | 1 | 0.01 | 0.0192 |
| GO:0045478 | fusome organization | 7 | 1 | 0.01 | 0.0192 |
| GO:0007282 | cystoblast division | 4 | 1 | 0 | 0.0204 |
| GO:0032403 | protein complex binding | 65 | 1 | 0.06 | 0.025 |
| GO:0090527 | actin filament reorganization | 2 | 1 | 0 | 0.029 |
| GO:0042537 | benzene-containing compound metabolic process | 13 | 1 | 0.01 | 0.0296 |
| GO:0022414 | reproductive process | 277 | 2 | 0.3 | 0.0336 |
| GO:0006807 | nitrogen compound metabolic process | 2585 | 6 | 2.81 | 0.0341 |
| GO:0003006 | developmental process involved in reproduction | 211 | 2 | 0.23 | 0.0347 |
| GO:0034440 | lipid oxidation | 10 | 1 | 0.01 | 0.0355 |
| GO:0051293 | establishment of spindle localization | 11 | 1 | 0.01 | 0.0369 |
| GO:0051295 | establishment of meiotic spindle localization | 2 | 1 | 0 | 0.0377 |
| GO:0019482 | beta-alanine metabolic process | 11 | 1 | 0.01 | 0.0399 |
| GO:0030261 | chromosome condensation | 16 | 1 | 0.02 | 0.0399 |
| GO:0042078 | germ-line stem cell division | 8 | 1 | 0.01 | 0.0406 |
| GO:0000922 | spindle pole | 13 | 1 | 0.02 | 0.0411 |
| GO:0045298 | tubulin complex | 30 | 1 | 0.04 | 0.0428 |
| GO:0022402 | cell cycle process | 224 | 2 | 0.24 | 0.0463 |
