# Supplementary material for: Evolution of Social Insect Polyphenism Facilitated by the Sex Differentiation Cascade
Source: PLoS Genet. 2016 Mar 31;12(3):e1005952. doi: 10.1371/journal.pgen.1005952 (PMC4816456; doi:10.1371/journal.pgen.1005952)
Supplement: S8 Table — Primers used for 3’ Rapid amplification of cDNA ends (RACE), in real-time quantitatitive PCR (qPCR) as housekeepers (HK) or targets (T), or for microsatellite analyses (MS). (DOCX) [file pgen.1005952.s008.docx]

**S8 Table**

| primer name | sequence (5’ 🡪 3’) | target (transcript-ID / scaffold, positions) | usage |
| --- | --- | --- | --- |
| RACE outer = dsx4_for4 | TTGATCTCAGAGGACACAAG | *Cobs_01393 (dsx)*, exon 2 | RACE +  qPCR (T) |
| RACE inner = Co_dsx_p3_for | GATGAACGAATTTGCCTCGGTC | *Cobs_01393 (dsx)*, exon 3 | RACE |
| dsx4_rev1 | GGATAGGAAACCAATGATGAT | *Cobs_01393 (dsx)*, exon 3 | qPCR (T) |
| 4for | CCTCGGACTTGAGACTAAACG | *Cobs_01393 (dsx)*, exon 4 | qPCR (T) |
| F5rev | GGCGAATATTTTTCAAATGACGAG | *Cobs_01393 (dsx)*, exon 5 | qPCR (T) |
| M5rev | GTTCTCACAACCATCGATGATG | *Cobs_01393 (dsx)*, exon 6 | qPCR (T) |
| RPL32_for | TCGCAGGCGTTTTAAGGGCCA | *Cobs_10346* (60S ribosomal protein L32) | qPCR (HK) |
| RPL32_rev | CTCCGAACGCAAGCGTGCACTA |  |  |
| RPS2_new_for | AAGCCATTCTGCGATGGCC | *Cobs_18295* (DNA-directed RNA polymerase II subunit RPB1, multi-copy gene) | qPCR (HK) |
| RPS2_new_rev | TCGAAGCCAACATGCTTAGCG |  |  |
| Y45F10D_JO1_for | CATCGGCGCGACGTCCAAGA | Cobs_04843 (iron-sulfur cluster assembly enzyme ISCU, mitochondrial) | qPCR (HK) |
| Y45F10D_JO1_rev | GCCCCCACCAGACCTGTTCC |  |  |
| Cobs_1.1_for-FAM | GGATCCCGAAATCAGCTAAAATAAA | scf0002  7,823,131 – 7,823,155  7,823,288 – 7,823,267 | MS |
| Cobs_1.1_rev | CGTATCAACTGGAAATTTTAGA |  |  |
| Cobs_8.3_for-FAM | TAACAGAGTTCCGTAGGTTT | scf0008  2,887,283 – 2,887,302  2,887,416 *–* 2,887,394 | MS |
| Cobs_8.3_rev | TGATGTTATCTCAATACTGGTCT |  |  |
| Cobs_8.4_for-HEX | GCTAAACGCAGTCACAGTCT | scf0008  3,226,875 - 3,226,894  3,227,032 – 3,227,008 | MS |
| Cobs_8.4_rev | TGTTATATCTCTTAAAAATTTGCAT |  |  |
