# Supplementary material for: Evolution of Social Insect Polyphenism Facilitated by the Sex Differentiation Cascade
Source: PLoS Genet. 2016 Mar 31;12(3):e1005952. doi: 10.1371/journal.pgen.1005952 (PMC4816456; doi:10.1371/journal.pgen.1005952)
Supplement: S9 Table — Apis mellifera (Amel), Nasonia vitripennis (Nvit), Cardiocondyla obscurior (Cobs), Acromyrmex echinatior (Aech) and Pogonomyrmex barbatus (Pbar) predicted proteins were accessed by BLASTp and tBLASTn analyses on http://hymenopteragenome.org/. Drosophila melanogaster (Dmel) proteins were accessed on http://flybase.org/blast/. (DOCX) [file pgen.1005952.s009.docx]

**S9 Table**

| species (database) | proteins |
| --- | --- |
| Dmel  (Flybase) | dsx (FBgn0000504) |
|  | dmrt99B (FBgn0039683) |
|  | dmrt93B (FBgn0038851) |
|  | dmrt11E (FBgn0030477) |
| Amel  (Amel_4.5_OGSv3.2_pep) | GB55036 |
|  | GB46471 |
|  | GB43847 |
|  | GB46747 |
| Nvit  (Nasonia Official Gene Set v1.2 Protein) | dsx: Genbank accession: NP_001155990 |
|  | NV17051 |
|  | NV23337 |
|  | no predicted transcript; chrUn: positions 3,401,206 – 3,401,096 |
| Cobs  (Cardiocondyla_ obscurior_1.4_ proteins) | Cobs_09254 (scf0029) |
|  | Cobs_18158 (scf0002) |
|  | Cobs_07724 (scf0049) |
|  | Cobs_01393 (scf0005) |
| Aech  (Aech_OGSv3.8_ proteins) | AECH26796 |
|  | AECH17342 |
|  | AECH12121 |
|  | no predicted transcript; scf162: positions 728,652 – 728,804 |
| Pbar  (proteins 1.2) | PB13071 |
|  | PB18113 |
|  | PB24480 |
|  | PB15498 |
