# Supplementary material for: Evolution of Social Insect Polyphenism Facilitated by the Sex Differentiation Cascade
Source: PLoS Genet. 2016 Mar 31;12(3):e1005952. doi: 10.1371/journal.pgen.1005952 (PMC4816456; doi:10.1371/journal.pgen.1005952)
Supplement: S3 Fig — (A) dsxF expression is higher in female (red triangles) than male halves (blue squares) of each individual, whereas dsxM expression is higher in male than female halves, with one exception (#10) (B). (DOCX) [file pgen.1005952.s012.docx]

**S3 Fig**
