# Supplementary material for: Evolution of Social Insect Polyphenism Facilitated by the Sex Differentiation Cascade
Source: PLoS Genet. 2016 Mar 31;12(3):e1005952. doi: 10.1371/journal.pgen.1005952 (PMC4816456; doi:10.1371/journal.pgen.1005952)
Supplement: S4 Fig — RNAseq data of larvae of all four morphs (EM = wingless, ergatoid males, WM = winged males, WO = workers, QU = queens, N = 7 each) were analyzed using DEXseq. Female castes show significant differences in expression of exons 3 and 7, male morphs show significant differences in all exons except exon 5. Letters indicate test statistics of pairwise Wilcoxon Tests with Benjamini-Hochberg correction for multiple testing (see S5 Table). (DOCX) [file pgen.1005952.s013.docx]

**S4 Fig**
