# Supplementary material for: Evolution of Social Insect Polyphenism Facilitated by the Sex Differentiation Cascade
Source: PLoS Genet. 2016 Mar 31;12(3):e1005952. doi: 10.1371/journal.pgen.1005952 (PMC4816456; doi:10.1371/journal.pgen.1005952)
Supplement: S5 Fig — Red values show bootstrap probabilities (bp); blue values show approximately unbiased p-values (au) from pvclust. Workers (WO) and queens (QU) are well separated, whereas one winged male (WM) clusters within the wingless, ergatoid males (EM) and one wingless male clusters within the winged males. (DOCX) [file pgen.1005952.s014.docx]

**S5 Fig**
