# Supplementary material for: Evolution of Social Insect Polyphenism Facilitated by the Sex Differentiation Cascade
Source: PLoS Genet. 2016 Mar 31;12(3):e1005952. doi: 10.1371/journal.pgen.1005952 (PMC4816456; doi:10.1371/journal.pgen.1005952)
Supplement: S6 Fig — Red values show bootstrap probabilities (bp); blue values show approximately unbiased p-values (au) from pvclust. (DOCX) [file pgen.1005952.s015.docx]

**S6 Fig**
