# Supplementary material for: Evolution of Social Insect Polyphenism Facilitated by the Sex Differentiation Cascade
Source: PLoS Genet. 2016 Mar 31;12(3):e1005952. doi: 10.1371/journal.pgen.1005952 (PMC4816456; doi:10.1371/journal.pgen.1005952)
Supplement: S7 Fig — DM domain-containing proteins of Drosophila melanogaster (Dmel), Apis mellifera (Amel), Nasonia vitripennis (Nvit), Acromyrmex echinatior (Aech), Pogonomyrmex barbatus (Pbar) and Cardiocondyla obscurior (Cobs) were aligned with MUSCLE. (DOCX) [file pgen.1005952.s016.docx]

**Figure S7: Alignment of the amino acid sequences of the DM domain (pfam00751).** DM domain-containing proteins of *Drosophila melanogaster* (Dmel), *Apis mellifera* (Amel), *Nasonia vitripennis* (Nvit), *Acromyrmex echinatior* (Aech), *Pogonomyrmex barbatus* (Pbar) and *Cardiocondyla obscurior* (Cobs) were aligned with MUSCLE.
